# Aneuploidy causes premature differentiation of neural and intestinal stem cells

**DOI:** 10.1038/ncomms9894

**Published:** 2015-11-17

**Authors:** Delphine Gogendeau, Katarzyna Siudeja, Davide Gambarotto, Carole Pennetier, Allison J. Bardin, Renata Basto

**Affiliations:** 1 Institut Curie, PSL Research University, CNRS UMR144, 12 rue Lhomond, Paris 75005, France; 2 Institut Curie, CNRS UMR3215, INSERM U934, Paris 75005, France

## Abstract

Aneuploidy is associated with a variety of diseases such as cancer and microcephaly. Although many studies have addressed the consequences of a non-euploid genome in cells, little is known about their overall consequences in tissue and organism development. Here we use two different mutant conditions to address the consequences of aneuploidy during tissue development and homeostasis in *Drosophila*. We show that aneuploidy causes brain size reduction due to a decrease in the number of proliferative neural stem cells (NSCs), but not through apoptosis. Instead, aneuploid NSCs present an extended G1 phase, which leads to cell cycle exit and premature differentiation. Moreover, we show that this response to aneuploidy is also present in adult intestinal stem cells but not in the wing disc. Our work highlights a neural and intestine stem cell-specific response to aneuploidy, which prevents their proliferation and expansion.

## Abstract (web summary)

It is unclear why certain tissues are more susceptible to the consequences of aneuploidy. Here, in *Drosophila*, Gogendeau *et al.* identify aneuploidy as the cause of lengthened G1 and premature differentiation in both neural and adult intestinal stem cells, which prevents cells with abnormal genomes from cycling.

## Graphical abstract

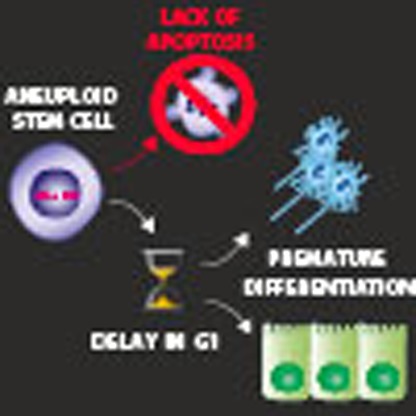

During development the precise coordination between cell proliferation, death and differentiation ensures the assembly of functional tissues and organs of the correct size. Regulation of cell cycle length, number and outcome of stem cell divisions allows the generation of cell lineages in a spatial and temporal manner. Central to this control is the maintenance of a diploid genome as genome imbalance can result in decreased cell fitness, impaired viability and lead to developmental disorders or ultimately to death^1^. A current challenge in the developmental biology field is to understand how rapidly dividing cells maintain a diploid genome and coordinate different types of cell cycle parameters that sustains stem cell self-renewal, cell proliferation and differentiation within a developing organism^2^.

Aneuploidy, the gain or loss of whole chromosomes, resulting from errors occurring during mitosis has been recognized as a major feature of human cancers since the pioneer work of Boveri^3^. However, aneuploid conditions can lead to other disorders such as growth defects and mental retardation: Down and Turner syndromes^4^,^5^, mosaic variegated aneuploidy (MVA)^6^ and microcephaly^7^. This raises the interesting question of whether aneuploidy and its consequences can predispose to different outcomes depending on cell type, developmental timing and age.

The *Drosophila* central nervous system is an excellent genetically tractable system to study the consequences of aneuploidy^8^,^9^. The central brain (CB) region contains neural stem cells (NSCs), also known as neuroblasts (Nbs) of embryonic origin that re-enter the cell cycle after a quiescence period during early larval stages^10^. Neuroblasts divide asymmetrically to self-renew and to produce a ganglion mother cell (GMC) which will divide once more before differentiating into neurons or glia. Asymmetric cell division and the generation of two daughter cells with distinct cell fates rely on the differential segregation of polarity and cell fate determinants coupled to correct spindle position along the polarity axis during metaphase^9^.

In flies, defects in centrosome biogenesis cause spindle mispositioning and tumour formation in transplantation assays^11^,^12^, while aneuploid mutations do not^11^. Aneuploid mutants die at the end of larval stages, showing that accumulation of aneuploidy is not compatible with metamorphosis and adult life^8^. In contrast to the observations made in the brain, aneuploidy in other proliferative tissue, such as the wing disc was found to be a tumour-initiating event^13^,^14^. In mice, deregulation of the levels of checkpoint proteins caused tumours in a tissue-dependent manner^15^,^16^. Aneuploid mice displayed higher incidence of lymphomas and lung tumours but lower frequency of chemically induced tumours when compared with controls. It is therefore essential to understand the reasons why aneuploidy in certain tissues is permissive to tumour initiation, while in others inhibits tumour formation.

Here we use the *Drosophila* brain to investigate the consequences of aneuploidy in brain homeostasis and the outcome of combining aneuploidy with a tumour-permissive condition, centrosome amplification. We show that aneuploidy decreases the tumourigenic potential of NSCs. In addition, we found that aneuploid NSCs do not die by apoptosis. Instead, aneuploid NSCs display G1 lengthening and undergo premature differentiation. Further, we show that adult intestine stem cells (ISCs) present the same type of response. Our work identifies an outcome of aneuploid NSCs and adult ISCs, which inhibits the proliferation and accumulation of abnormal karyotypes in two proliferative tissues.

## Results

### Generating non-euploid cells in fly brains

To characterize the outcome of aneuploid NSCs during brain development in *Drosophila,* we used a previously described mutant *SakOE,mad2* where the centriole kinase Sak, the Plk4 fly ortholog, is overexpressed (*SakOE*) leading to centrosome amplification^12^ in the absence of the checkpoint protein Mad2 (ref. 17)*. SakOE* third-instar larval (L3) NSCs (called neuroblasts-Nbs) of the CB possess extremely efficient clustering mechanisms of extra centrosomes and generate low aneuploidy levels^12^. *mad2* mutants are viable and fertile since reduction of the mitotic timing does not affect cell division in flies^17^.

**Figure 1:**
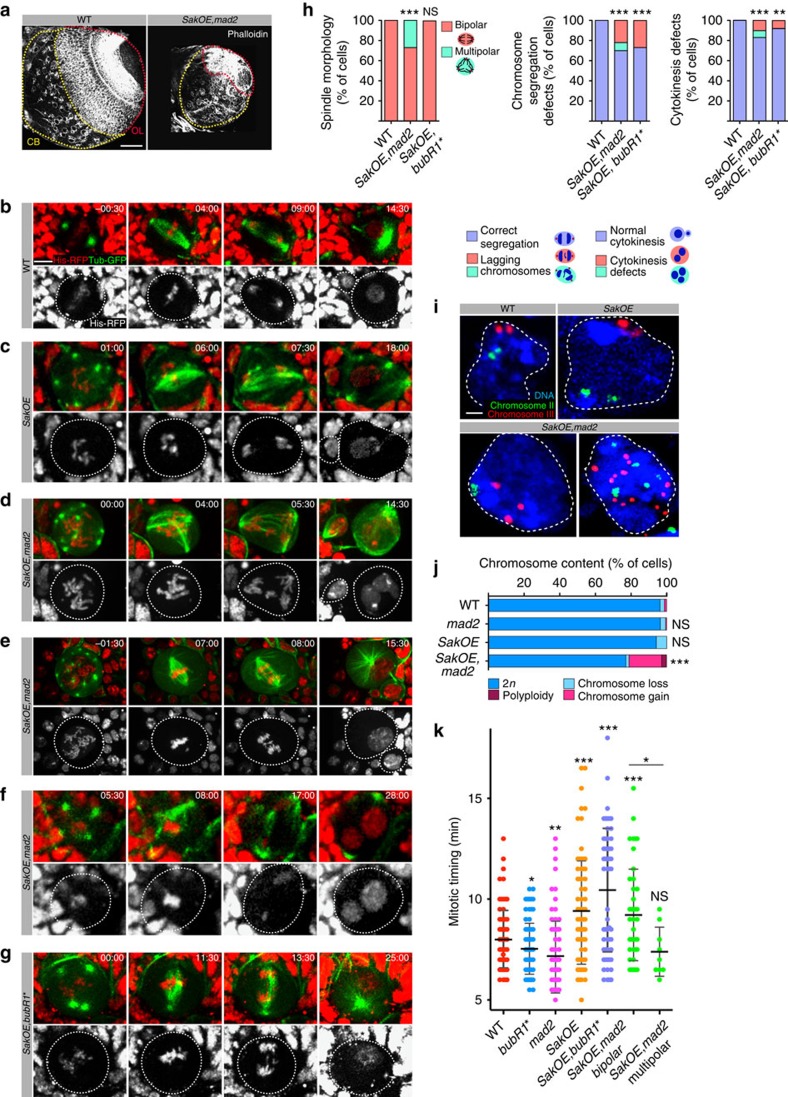
Characterization of aneuploid brains. (**a**) Phalloidin staining of wild-type (WT) (left) and *SakOE,mad2* (right) brain lobes. In the WT lobe both the CB, highlighted by the yellow dashed line, and optic lobe (OL), highlighted by the red dashed line appear highly organized, while *SakOE,mad2* lobes appear smaller and disorganized. Scale bar, 50 μm. (**b**–**g**) Stills of time-lapse movies of mitotic neuroblasts (Nbs) expressing Histone 2B-RFP and Tubulin-GFP, in red and in green, respectively. (**b**) Wild-type Nb with two centrosomes forms a bipolar spindle and divides asymmetrically to give rise to two cells. (**c**) Sak*OE* Nb with at least five centrosomes that form a bipolar spindle due to centrosome clustering and inactivation generating two daughter cells. (**d**) *SakOE,mad2* Nb with at least ten centrosomes and increased chromosome number. Not all centrosomes cluster and the cell undergoes a tripolar division. (**e**) *SakOE,mad2* Nb with increased chromosome number and at least 15 centrosomes that cluster to form a bipolar spindle. Lagging chromosomes are noticed during anaphase. (**f**) *SakOE,mad2* Nb divides in a bipolar way, but presents defects in cytokinesis. (**g**) SakOE, bubR1* (bubR1^*ΔKEN*^ in *bubR1* mutant background) Nb divides in a bipolar way but shows extra lagging chromatids during anaphase. Scale bar, 3 μm. (**h**) Graph bars showing the quantification of mitotic defects in WT (*n*=110), *SakOE,mad2* (*n*=166) and SakOE, bubR1* (*n*=64) Nbs, considering spindle morphology (left), chromosome segregation defects (middle) and cytokinesis defects (right). Statistical significance (SS) was determined using Fisher's exact test. ns, not significant, ***(*P*<0.0001), **(*P*=0.0061). (**i**) Fluorescent *in situ* hybridization with chromosomes II and III probes (green and red) in WT, *SakOE,* and *SakOE,mad2* Nbs. Scale bar, 2 μm. (**j**) Graph bars showing the quantification of FISH in WT (*n*=227 cells), *mad2* (*n*=166 cells)*, SakOE* (*n*=153 cells) and *SakOE,mad2* (*n*=109 cells) brains. (**k**) Dot plot chart showing the time spent in mitosis measured as the time elapsed between nuclear envelope breakdown and anaphase onset. Each dot represents a cell. Time is in minutes and the line represents the mean and the error bars the s.d. Statistical significance was assessed by unpaired *t*-test. *(0.01<*P*<0.05), **(0.001<*P*<0.01) and ***(*P*<0.001).

*SakOE,mad2* mutants die at pupal stage and at larval stages present tissue size defects such as smaller imaginal discs, similarly to other aneuploid mutants^8^. Analysis of mid L3 *SakOE,mad2* brains revealed a drastic reduction of both the neuroepithelium and CB regions, which appeared to be very disorganized (Fig. 1a).

To gain information on the mitotic defects generated in the presence of extra centrosomes and lack of spindle assembly checkpoint (SAC), we followed, by time-lapse spinning disc microscopy, mitosis of L3 Nbs of the CB expressing Tubulin-GFP and Histone 2B (H2B)-RFP. All wild-type (WT) Nbs contained two centrosomes and divide in a bipolar way, generating two cells of different size and fate^9^,^10^ (Fig. 1b and Supplementary Movie 1). *SakOE* Nbs that contained extra centrosomes also divided in a bipolar way, as observed previously^12^ (Fig. 1c and Supplementary Movie 2). In *SakOE,mad2* Nbs with extra centrosomes, however, several cell division defects were noticed such as multipolar divisions, in 27% (*n*=46 out of 166) of the Nbs (Fig. 1d,h and Supplementary Movie 3). Furthermore, in 26% (*n*=26 out of 90) of *SakOE,mad2* Nbs, bipolar anaphases with lagging chromosomes were observed (Fig. 1e,h and Supplementary Movie 4), suggesting that chromosomes were mis-segregated even in clustered bipolar divisions, similarly to what has been described in tissue culture cells^18^,^19^. Failure in cytokinesis was observed in 17% (*n*=35 out of 196) of Nbs (Fig. 1f,h and Supplementary Movie 5). Cytokinesis defects were observed both in cells that divide bipolarly (10%) and multipolarly (7%) and frequently this correlated with the presence of lagging chromatids at the cytokinesis furrow. Importantly, in WT, *SakOE* or *mad2* Nbs, these defects were never observed.

Using fluorescence *in situ* hybridization (FISH) we found that 19.1% (*n*=109) of *SakOE,mad2* Nbs, were aneuploid (1.8% with loss of one chromosome, 11.8% with gain of one chromosome and 5.5% with gain or loss of two or more than two chromosomes), while 1.8% were polyploid (Fig. 1i,j). Although low levels of chromosome gain and losses were also noticed in WT, *mad2* and *SakOE* brains (3.8%, 3.6% and 5.9%, respectively), these Nbs did not show polyploidy or gain/loss of more than one chromosome (Fig. 1i,j).

It has been shown recently that chromosome mis-segregation causes structural DNA aberrations^20^,^21^. We analysed DNA damage in *SakOE,mad2* brains measured by damaged dependent phosphorylation of the Histone variant H2Av (γ-H2Av). As control of γ-H2Av antibody, we incubated the brains with the DNA synthesis inhibitor, aphidicolin to induce replication stress and consequently accumulate double-strand breaks. Incubation of WT brains with aphidicolin for 30 min was sufficient to detect an increase in γ-H2Av-positive cells in the CB (Supplementary Fig. 1A,B); however, untreated WT and *SakOE,mad2* brains showed comparable levels of γ-H2Av-positive cells. We conclude that chromosome mis-segregation in *SakOE,mad2* brains does not result in DNA damage.

The SAC guarantees mitotic fidelity by delaying anaphase onset until all chromosomes are correctly attached to microtubules^22^. Extra centrosomes are known to cause mitotic lengthening, which can serve as an advantage to cells that contain extra centrosomes^12^,^23^. To understand why some spindles manage to be bipolar while others maintain a multipolar status, we decided to separate mitotic timing from checkpoint function^24^. To this end, we used a mutant version of BubR1 (ΔBubR1^ΔKen^ in the *bubR1* mutant background, that we will refer to as BubR1*), which has been shown to perturb BubR1 checkpoint activity while it did not affect mitotic timing^25^. *SakOE*, BubR1* mitotic Nbs analysis revealed that, even if lagging chromosomes (28%) and cytokinesis defects (8%) were identified in bipolar divisions, we never observed multipolar divisions (*n*=64) (Fig. 1g,h and Supplementary Movie 6). We quantified the time in mitosis as the time elapsed between nuclear envelope breakdown and anaphase onset. WT Nbs spent on average 7.9±0.2 min in mitosis, while *mad2* and *BubR1* Nbs spent 7.2±0.2 and 7.5±0.1 min in mitosis. *SakOE* Nbs underwent slower mitosis, consistent with previous findings (9.4±0.3 min)^12^. Mitotic timing was also increased in *SakOE*, BubR1* (9.8±0.5 min) and in bipolarly dividing *SakOE,mad2* Nbs (9.2 ±0.3 min). In contrast, *SakOE,mad2* Nbs showing multipolar divisions spent on average less time in mitosis (7.4±0.4 min) (Fig. 1k). These results show a correlation between mitotic timing and bipolar status in cells that contain extra centrosomes. As shown in other cell types^23^,^26^, mitotic timing but not the activity of the SAC *per se* contributes to bipolar spindle formation in the presence of extra centrosomes.

### Aneuploid brains contain fewer neuroblasts

**Figure 2:**
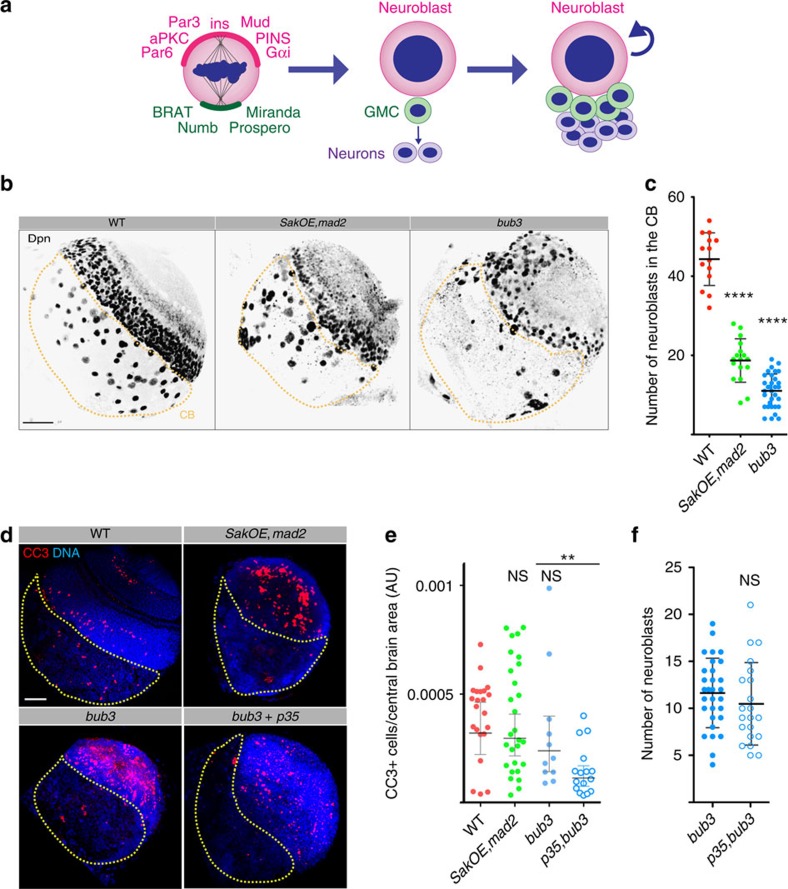
Apoptosis is not the cause of Nbs loss in aneuploid brains. (**a**) Schematic representation of L3 type I Nbs asymmetric divisions. The larger pink cell represents one Nb that asymmetrically segregates polarity cues and cell fate determinants. Spindle positioning along the polarity axis generates two daughter cells of unequal fate and size. The larger self-renewing cell, inherits the apical Par complex and Insc/Pins/Mud/Gαi complexes and maintains Nb identity whereas the small green daughter cell (the GMC) receives Numb, Pros and Brat, which promote differentiation and suppress self-renewal. The GMC divides once more to generate two terminally differentiated neurons or glial cells (purple cells). (**b**) Pictures of WT, *SakOE,mad2* and *bub3* L3 brain lobes stained for Deadpan (Dpn) a Nb marker. Aneuploid brains show a reduced number of Nbs in the CB region, highlighted by the yellow dashed line. Scale bar, 50μm. (**c**) Dot plot chart showing the quantification of Nbs number (Dpn^+^ cells) in WT (red dots, *n*=14 lobes), *SakOE,mad2* (green dots, *n*=17 lobes) and *bub3* (blue dots, *n*=32 lobes) CBs. Each dot represents a brain lobe. The line represents the mean and the error bars the s.d. Statistical significance (SS) was assessed by an unpaired *t*-test. ****(*P*<0.0001). (**d**) Pictures of WT, *SakOE,mad2*, *bub3* and *p35,bub3* L3 brain lobes stained with antibodies against cleaved caspase 3 (CC3, shown in red). DNA is shown in blue. The yellow dashed line marks the CB region. Scale bar, 50 μm. (**e**) Dot plot chart showing the quantification of the ratio between CC3 positive cells per CB area in WT (red dots), *SakOE,mad2* (green dots), *bub3* (blue dots) and *p35, bub3* (blue circles) brains. Each dot represents the rate measured in each brain lobe. The line represents the mean and the error bars the s.d. SS**** was assessed by an unpaired *t*-test. **(*P*=0.005). (**f**) Dot plot chart showing the quantification of Nbs number in *bub3* (full blue circles) and *p35,bub3* brain lobes (empty blue circles) where apoptosis has been inhibited. The line represents the mean and the error bars the s.d. SS was assessed by an unpaired *t*-test.

*SakOE,mad2* brains appeared smaller than WT brains (Fig. 1a) and we investigated the CB cell population. At early L3, previously quiescent type I Nbs re-enter the cell cycle and divide asymmetrically several times to self-renew^9^,^10^,^27^ (Fig. 2a) generating around 100 daughter cells^28^,^29^. *SakOE,mad2* brains contained fewer Nbs positive for the Deadpan (Dpn) (Dpn^+^) marker when compared with WT brains (Fig. 2b,c). We then extended our analysis to the previously characterized aneuploid mutant with normal centrosome content, *bub3* (refs 30, 31, 32). We used *bub3* mutants because they die at the same developmental stage as *SakOE,mad2* and *bub3* Nbs display similar levels of aneuploidy although we did not detect polyploidy (Supplementary Fig. 2A,B). We characterized *bub3* mitoses and found that all mitoses were bipolar (see below) and that frequently lagging chromosomes could be seen, as described before^31^. Similarly to *SakOE,mad2* brains, we did not detect an increase in γ-H2Av-positive nuclei (Supplementary Fig. 2C,D). Importantly, we found that just like in *SakOE,mad2* brains, the number of Nbs was reduced in *bub3* mutant brains (Fig. 2b,c).

Since apoptosis is a common fate of aneuploidy cells^7^,^13^,^14^,^33^, we ascertained whether reduced number of Nbs was due to apoptosis. We investigated the presence of cleaved caspase 3-positive cells (CC3^+^) in the CB. We found that the number of CC3^+^ Nbs did not increase significantly in both *SakOE,mad2* and *bub3* CBs (Fig. 2d,e); however, it was detected in mutant optic lobes (OL) (Fig. 2d), similarly to what has been recently shown in *asp* mutant OL^34^. Interestingly, these results show that two different regions of the brain respond differently to aneuploidy. Importantly, suppression of apoptosis, using the pan-caspase p35 inhibitor did not rescue the decrease in Nb number of aneuploid brains (Fig. 2d–f). We controlled the efficiency of p35 in inhibiting apoptosis in the posterior compartment of *bub3* mutant wing discs, using the engrailed-Gal4 (eng-Gal4) promoter. As described previously, suppression of apoptosis by expression of p35 in *bub3* mutant wing discs resulted in highly abnormal discs (Supplementary Fig. 3) due to the accumulation of aneuploid cells^13^,^14^,^32^. We conclude that the reduced Nb number found in aneuploid mutant brains cannot be explained by apoptosis.

Certain brain Nb lineages do not seem to undergo apoptosis at least during larval stages^35^, but apoptosis is used to inhibit the proliferation of other lineages such as Mushroom body Nbs during pupal stages^36^. To test whether larval Nbs can undergo apoptosis in response to other insults, we treated WT brains with aphidicolin, to induce DNA damage (Supplementary Fig. 1A,B). We observed CC3^+^ Nbs, (Supplementary Fig. 4), showing that even if Nbs can undergo apoptosis in response to DNA damage, aneuploidy does not trigger this cell death response.

We next investigated whether another type of cell death, necrosis, was responsible for Nb loss seen in aneuploidy mutants. Recently, the Fzy protein, the Cdc20 APC/C activator homologue, has been implicated in necrosis^37^. Interestingly, mutations in *fzy*, also lead to premature Nb loss and *fzy* Nbs accumulated several stress markers, showed loss of membrane integrity and upregulation of p53 (ref. 37). We first analysed Fzy levels by western blot in *SakOE,mad2* and *bub3* brains, and found that these were comparable to WT brains (Supplementary Fig. 5A). We then analysed stress markers such as the presence of ubiquitin-conjugated protein aggregates, mitochondria aggregation and ROS accumulation. Aneuploid brains showed an increase in the percentage of cells positive for ubiquitin-conjugated protein aggregates, when compared with WT brains (Supplementary Fig. 5B,C) (5.1% in *SakOE,mad2*, 22.5% in *bub3 and* 2.0% in WT), however not to the same extent as *fzy* mutants (40%)^37^. In contrast to *fzy* mutants, however, mitochondria aggregation or accumulation of ROS in aneuploid brains, were comparable to WT brains (Supplementary Fig. 5D–G). In addition, we never observed karyolysis or loss of membrane integrity (Supplementary Fig. 5H) as reported in *fzy* mutants and in other cells undergoing necrosis^38^. We next analysed the contribution of p53 to Nb loss in aneuploid brains using a p53 GFP reporter (p53RGFP)^39^. We identified on average eight GFP-positive cells in *SakOE,mad2* CB, while WT brains did not show p53 recruitment. However, in the large majority of the cases, the cells that appeared positive were not Nbs (Dpn^−^), but rather GMCs, neurons (Pros^+^ or Elav^+^, respectively) or lacked any of these markers (Supplementary Fig. 6A–C). Further, inhibition of p53 activity, either using a dominant negative version (R155H) or p53^RNAi^^40^ exclusively in Nbs, using a specific Nb driver, did not rescue the Nb loss phenotype (Supplementary Fig. 6D–G). All together, these results show that reduction of the number of CB Nbs in aneuploid brains is not explained by the same mechanism as in *fzy* mutants. In agreement, *fzy* mutants did not show chromosome mis-segregation defects^37^.

### Aneuploid brains undergo premature differentiation

**Figure 3:**
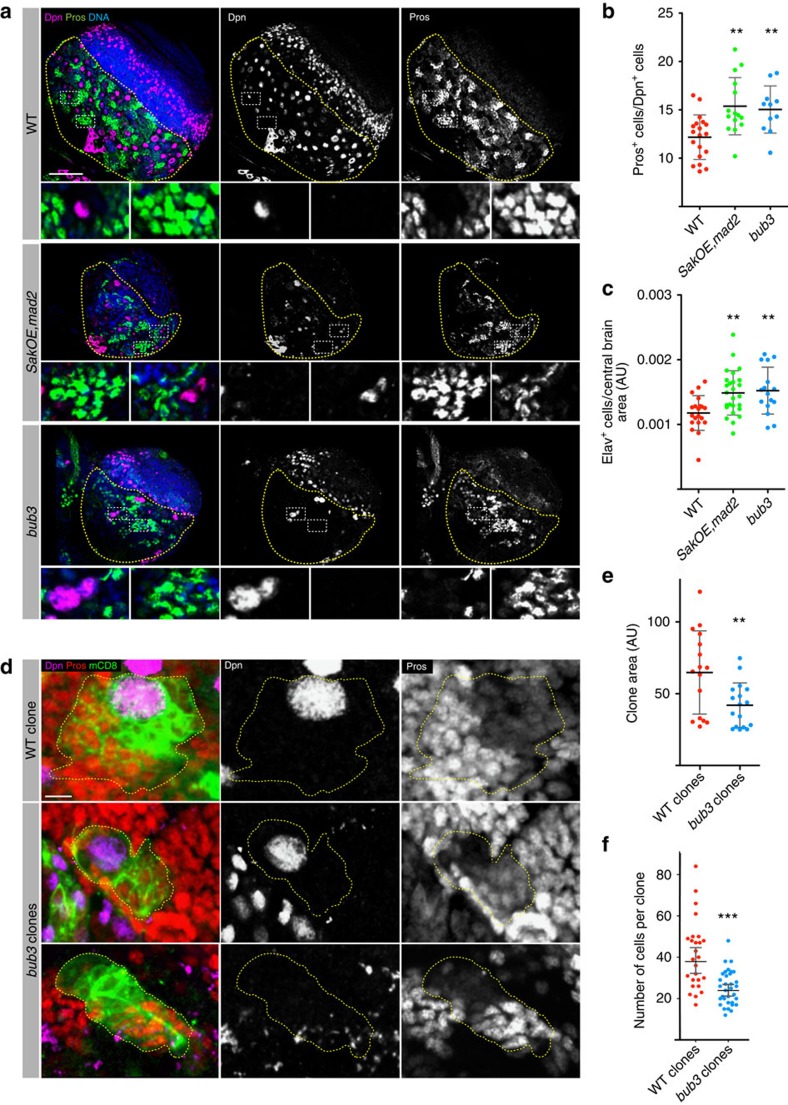
Self-renewal and differentiation in aneuploid brains. (**a**) Pictures of WT, *SakOE,mad2* and *bub3* brains stained for Dpn (middle and in purple to label Nbs) and Prospero (Pros) (right, in green to label GMCs). DNA is shown in blue. The yellow dashed line marks the CB region. Scale bar, 50 μm. (**b**) Dot plot chart showing the quantification of the ratio between nuclear Pros^+^ (GMCs) and Dpn^+^ (Nbs) in WT (red dots, *n*=19 lobes), *SakOE,mad2* (green dots, *n*=15 lobes) and *bub3* (blue dots, *n*=11 lobes) brains. Each dot represents the ratio from one brain lobe. The line represents the mean and the error bars the s.d. The raw data of quantifications are given in Supplementary Table 1. Statistical significance (SS) was assessed by an unpaired *t*-test. **(*P*<0.01). (**c**) Dot plot chart showing the quantification of the ratio between Elav^+^ (neurons) per CB area in WT (red dots, *n*=20 lobes), *SakOE,mad2* (green dots, *n*=26 lobes) and *bub3* (blue dots, *n*=16 lobes) brain lobes. Each dot represents the ratio from one brain lobe. The line represents the mean and the error bars the s.d. The corresponding pictures and the raw data of quantification are shown in Supplementary Fig. 7 and Supplementary Table 2. Statistical significances were assessed by an unpaired *t*-test. **(*P*<0.01). (**d**) Pictures of WT (top) and *bub3*^RNA*i*^ (middle and bottom) clones expressing mCD8 (shown in green to label the clone) and stained for Dpn (labelling Nbs, middle and in purple in the merge panel) and for Pros (labelling GMCs, right panel and in red in the merge channel). The bottom picture shows a clone that comprises a large cell not expressing Dpn and several GMCs. Scale bar, 5 μm. (**e**) Dot plot chart showing the quantification of clone area in WT (red dots) and *bub3*^RNAi^ (blue dots) clones. Each dot represents a clone. The line represents the mean and the error bars the s.d. Statistical significance was assessed by an unpaired *t*-test. **(*P*=0.0078). (**f**) Dot plot chart showing the quantification of number of cells per clone in WT (red dots) and *bub3*^RNA*i*^(blue dots) clones. Each dot represents a clone. The line represents the mean and the error bars the s.d. Statistical significance was assessed by unpaired *t*-test. ***(*P*=0.0006).

Since loss of Nbs in aneuploid brains is not caused by apoptosis or necrosis, we then tested whether these brains were undergoing premature differentiation. We stained L3 brains with Dpn, Prospero (Pros) and Elav antibodies to label Nbs, GMCs and neurons, respectively. Interestingly, the ratio between GMCs and Nbs was increased in *SakOE,mad2* and *bub3* brains when compared with WT (Fig. 3a,b, and Supplementary Table 1) in staged mid L3 larvae. In addition, the density of neurons (Elav^+^ cells per CB area) was also increased in aneuploid mutants when compared with WT (Fig. 3c, Supplementary Fig. 7 and Supplementary Table 2).

We then performed a clonal analysis in mid L3 brains to ascertain self-renew capacity and Nb progeny of WT and *bub3* mutant clones. Using the flip-out strategy^41^, clones were induced in staged L2 larvae by heat shock. After 48 h, brains were fixed and analysed. WT clones occupied a larger area (Fig. 3d,e) and contained ∼41±3.3 cells (*n*=26 clones from nine brains), while *bub3* aneuploid clones contained ∼25±1.5 cells (*n*=33 clones from eight brains) (Fig. 3f). Occasionally clones without any large Dpn^+^ cell (Fig. 3d) were also seen.

### Aneuploid brains display a G1 lengthening

**Figure 4:**
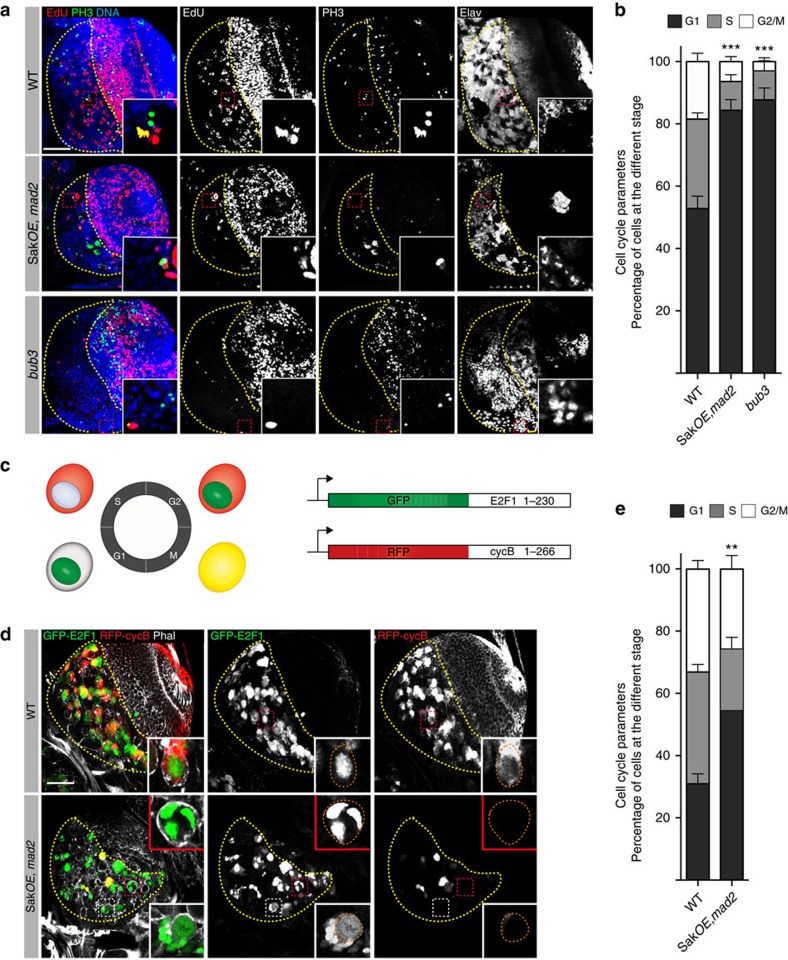
Aneuploid brains present an extended G1 phase. (**a**) Pictures of WT (upper panel), *SakOE,mad2* (middle panel) and *bub3* (lower panel) brains stained for EdU (5-ethynyl-2'-deoxyuridine) (second panel, shown in red in the merged panel), PH3 (third panel, shown in green in the merged panel) and Elav (right panel) labelling cells in S-phase, in M phase and neurons respectively. The CB is marked by the yellow dashed line. Scale bar, 50 μm. (**b**) Graph bar showing the quantification of cell cycle phase distribution in WT (4830 cells from eight lobes) *SakOE,mad2* (3220 cells from 10 lobes) and *bub3* (2973 cells from eight lobes) brains. Among the non-Elav cells, percentages of each cell cycle phases were estimated: EdU^+^: cells in S phase, EdU^+^PH3^+^ or PH3^+^: cells in G2/M Edu^−^,PH3^−^: cells in G1. In the CB, aneuploid brains present a higher percentage of cells in G1. Unpaired *t*-test. ***(*P*<0.001). (**c**) Diagram of the fluorescence ubiquitination cell cycle indicator (FUCCI) system. Fusions to GFP-E2F and RFP- Cyclin B are used as indicators of cell cycle progression^50^. G1 cells show nuclear green signal, S-phase cells show red cytoplasmic signal, and G2/M cells show green and red signals in the nucleus and cytoplasm respectively. M-phase cells round up and appear yellow. (**d**) Pictures of WT (top) and *SakOE,mad2* (bottom) brain lobes expressing the FUCCI system. The insets show G1 cells. In *SakOE,mad2*, the large cell with three nuclei probably resulted from cytokinesis defects described in Fig. 1. This type of cell is never seen in WT. Scale bar, 50 μm. (**e**) Graph bar showing the quantification of cell cycle phase distribution in WT (280 cells from 12 lobes) *SakOE,mad2* (261 cells from 18 lobes) brains. Unpaired *t*-test. **(*P*=0.0092).

Accumulating evidence showed a correlation between the length of the cell cycle and proliferation/differentiation capacity. Indeed, some stem cells undergo shorter cell cycles than their differentiating progeny^42^,^43^,^44^. Moreover, cell fate decisions have been postulated to be dependent on cell cycle progression^45^ and G1 lengthening is sufficient to promote neurogenesis of mouse neuronal progenitors^46^,^47^,^48^. Aneuploid yeast strains showed an extended G1 phase^49^ and we decided to determine if this was also the case in aneuploid mutant brains. We performed 2 h 5-ethynyl-2'-deoxyuridine (EdU) incorporation assays and manually counted the number of cells in S-phase (EdU positive cells -EdU^+^), in mitosis (PH3 positive cells-Ph3^+^) and all the nuclei. In addition, we also took into account the number of neurons (Elav^+^) to distinguish between cycling cells in G1 (EdU^−^, PH3^−^, Elav^−^) and terminally differentiated cells (EdU^−^, PH3^−^, Elav^+^) (Fig. 4a). Cells positive for EdU and PH3 were considered in the G2/M category, as cells that had during the 2 h EdU incubation period exit S-phase and progressed to mitosis. In WT brains, several EdU^+^ and PH3^+^ nuclei could be noticed (Fig. 4a,b) and our analysis shows that 53% of the cells were in G1 (*n*= 4830 cells from eight brain lobes Fig. 4b). In *SakOE,mad2 and bub3* brains, however, the number of EdU^+^ and PH3^+^cells was severely reduced (Fig. 4a,b). Importantly, more cells were arrested in G1, 84% (*n*=3220 cells from 10 lobes) in *SakOE,mad2* brains and 88% (*n*=2973 cells from eight lobes) in *bub3* (Fig. 4b).

To further investigate the distribution of aneuploid cells along the cell cycle, we used the fly-FUCCI (fluorescent ubiquitination-based cell cycle indicator) (Fig. 4c) (ref. 50). Analysis of WT brains showed that 30.9±3.2% of Nbs were in G1, 35.9±2.4% in S and 33.2±2.7% G2/M (*n*=280 Nbs from 12 lobes). In *SakOE,mad2* brains, a significant increase in G1 could be noticed (54.6±6.5% and of cells in G1, *n*=261 Nbs from 18 lobes) (Fig. 4d,e). Our results suggested an extended G1 in *SakOE,mad2* brains.

These results are in agreement with our live imaging observations of mid L3 brains. While in WT brains we can frequently follow consecutive Nb cell divisions due to the fast cell cycle of these cells (19 out of 93 Nbs re-enter mitosis), this event was very rare in *SakOE,mad2* brains (9 out of 127), suggesting that interphase was prolonged in aneuploid brains.

The Cdk inhibitor p27/dacapo is associated with terminal divisions and differentiation^51^. We investigated the role of Dacapo in premature differentiation, but found no consequence when dacapo levels were decreased (Supplementary Fig. 8B). We also tested the involvement of the p38 stress kinase, a known inhibitor of proliferation of aneuploid cells^33^, but found no effect (Supplementary Fig. 8A).

### Aneuploid neuroblasts differentiate prematurely

**Figure 5:**
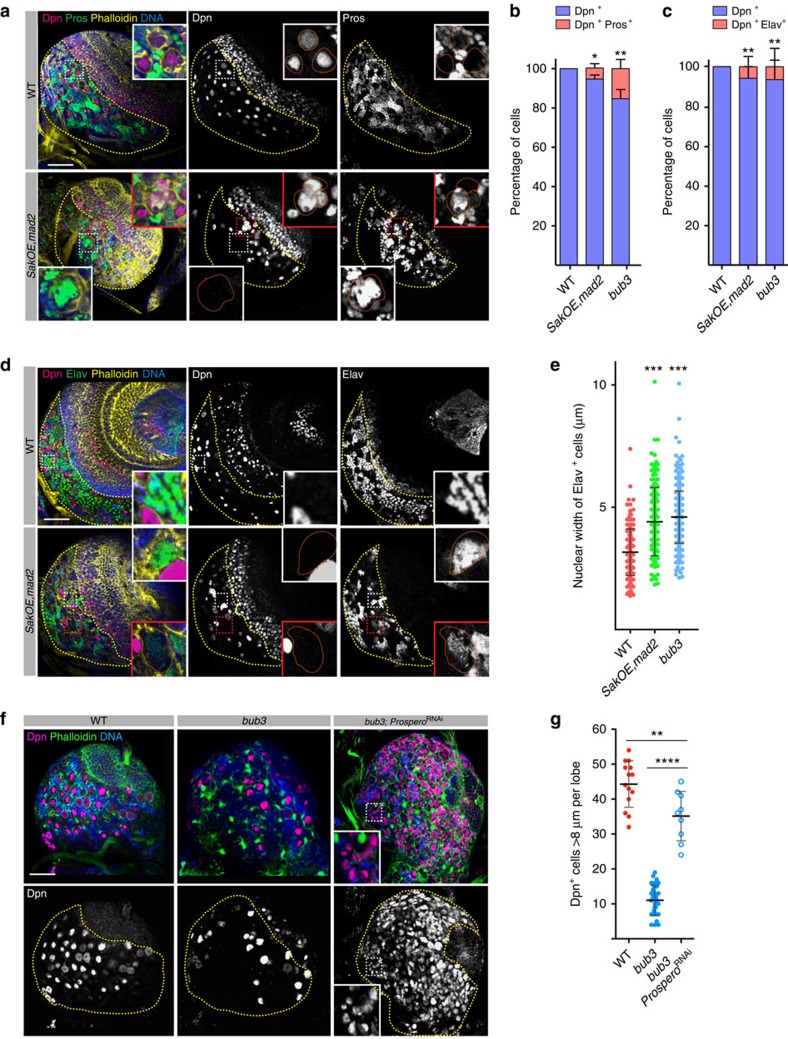
Aneuploid Nbs undergo premature differentiation in Pros-dependent manner. WT and *SakOE,mad2* brain pictures stained with Dpn (purple, middle panel), Pros (green, right panel), phalloidin is in yellow and DNA in blue. The yellow dashed line labels the CB. The large nuclei seen in the bottom inset might have resulted from cytokinesis defects, as described in Fig. 1. Scale bar, 50 μm. (**b**,**c**) Graph bars showing the percentage of Dpn^+^ and Dpn^+^, Pros^+^ cells in WT (525 cells/12 lobes), *SakOE,mad2* (324 cells/16 lobes) and *bub3* (103 cells/11 lobes) in (**b**) and the percentage of Dpn^+^ and Dpn^+^, Elav^+^ cells in WT (192 cells/4 lobes), *SakOE,mad2* (277 cells/ 16 lobes) and *bub3* (118 cells/10 lobes) in (**c**). Statistical significance determined using Fisher's exact test: in b- **(*P*=0.0012 *SakOE,mad2* and 0.0034 *bub3*), in c- *(*P*=0.024) and **(*P*=0.0012). (**d**) WT and *SakOE,mad2* brain pictures stained with Dpn (purple, middle panel) and Elav (green, right panel), phalloidin is in yellow and DNA in blue. The yellow dashed line labels the CB. Cells with large nuclei showing Elav accumulation can be seen (insets) in *SakOE,mad2* brains, but never in the WT. Scale bar= 50 μm. (**e**) Dot plot showing the nuclear width of Elav^+^ nuclei in WT (189 cells/6 lobes), *SakOE,mad2* (216 cells/11 lobes) and *bub3* (433 cells/5 lobes). The line represents the mean and the error bars the s.d. Statistical significance determined using an unpaired *t*-test. ***(*P*<0.0001). (**f**) WT, *bub3* and *bub3, Pros*^RNA*i*^ brain pictures stained with Dpn (purple and bottom panels), phalloidin is in green and DNA in blue. Scale bar, 50 μm. Since decrease in Pros levels causes reversion of differentiating daughters to Nb like cells^58^, small nuclei expressing the stem cell marker Dpn are present (inset). (**g**) Dot plot showing the number of Dpn^+^ nuclei with diameter >8 μm, to distinguish between previous GMCs and Nbs, in WT (red circles, 14 lobes), *bub3* (full circles, 32 lobes) and *bub3, Pros*^RNA*i*^ (empty circles nine lobes). The line represents the mean and the error bars the s.d. Statistical significance determined using an unpaired *t*-test. **(*P*=0.0048) and ****(*P*<0.0001).

Larval Nb size is progressively reduced at the end of larval stages throughout pupation^9^,^28^,^52^. We reasoned that if aneuploid Nbs were undergoing premature differentiation, the committed progenitors and resulting neurons might show increased nuclear size. In WT Nbs, the transcription factor Pros is sequestered by the adaptor Miranda (Mira), which is normally cortically localized. During anaphase, Mira is segregated to the daughter GMC, carrying Pros. Mira is subsequently degraded and Pros translocates to the nucleus to initiate its transcriptional dependent response^53^. In WT Nbs, Pros was never seen associated with the Nb nucleus (Fig. 5a,b) and was localized to the small GMC nucleus. In *SakOE,mad2* and *bub3* mutant brains; however, 5.6±2.2% (*n*=360 Nbs from 16 lobes) and 15.3±4.1% (103 Nbs from 11 lobes) of Nbs showed co-localization of Dpn and Pros (Fig. 5a,b), respectively. In addition large Pros^+^ nuclei could also be noticed, which were never seen in the WT CB (Fig. 5a,b). In WT GMCs Mira degradation allows Pros release into the nucleus^53^. We then investigated whether abnormal Pros nuclear localization, seen in Dpn^+^ Nbs from aneuploid brains, correlated with alteration in Mira levels. Even if 30.5% (*n*= 42 Nbs from 10 lobes) of Dpn^+^,Pros^+^ Nbs showed decreased Mira levels in *bub3* mutant brains, we also observed 69.5% of Dpn^+^,Pros^+^ Nbs where Mira levels were comparable to WT brains (Supplementary Fig. 9A,B). We concluded that if Mira degradation might contribute to Pros nuclear accumulation in the nucleus, other Mira independent mechanisms also participate in this process.

We then analysed the neuronal marker Elav. In *SakOE,mad2* and *bub3* Nbs, 5.2% (*n*=277 Nbs, from 16 lobes) and 6% (*n*=118 Nbs, from 10 lobes) appeared positive for both Dpn and Elav, which were never seen in the WT (Fig. 5c, *n*=192 Nbs, from four brain lobes). In addition, in the WT CB, Elav^+^ nuclei measure on average 3.2±0.1 μm (*n*=189 cells from six lobes), whereas larger Elav^+^ nuclei could be noticed in *SakOE,mad2* and *bub3* mutants CB, (Fig. 5d), and the average size was increased when compared with WT (4.4±0.09 μm, *n*= 216 cells from 11 lobes in *SakOE,mad2* and 4.6± 0,05 μm, *n*=433 cells in *bub3*, Fig. 5e). All together, these results show that aneuploid Nbs acquire GMC and neuronal markers, which probably lead to premature differentiation.

We then tested the role of Pros in premature differentiation as this transcription factor represses genes required for self-renewal while it activates genes necessary for differentiation^29^,^54^. We knocked down Pros exclusively in Nbs, using the Worniu-Gal4 driver. Since GMC maintenance requires Pros^54^ to distinguish between the consequences of Pros depletion in Nbs and GMCs, we took into account only Nbs with nuclear diameter superior to 8 μm as described previously^55^. Analysis of mid L3 brains, showed an increase in the number of CB Nbs (Fig. 5f,g). In addition, we also tested whether decreasing Pros levels would also result in similar rescue. For this purpose we analysed *bub3* mutant or *bub3*^RNA*i*^ brains in the *pros* heterozygous background. As before, a significant (using a Fischer's exact test) increase in the number of Dpn^+^ cells displaying >8 μm diameter, were detected (Supplementary Fig. 9C–F). Our results suggest that the premature differentiation seen in aneuploid brains recapitulates cell cycle exit and the normal differentiation of late L3 WT brains in a Pros-dependent manner.

### Aneuploidy in other tissues

To determine the fate of aneuploid cells in other tissues than the brain, we analysed aneuploid wing discs, which are highly proliferative non-stem epithelial cells and aneuploid intestinal stem cells of the adult midgut (ISCs). In contrast to CB Nbs, *SakOE,mad2* wing disc cells showed similar cell cycle parameters when compared with WT wing disc cells (Supplementary Fig. 10A,B). Furthermore, these discs showed high levels of apoptosis, in agreement with published results^13^,^14^.

**Figure 6:**
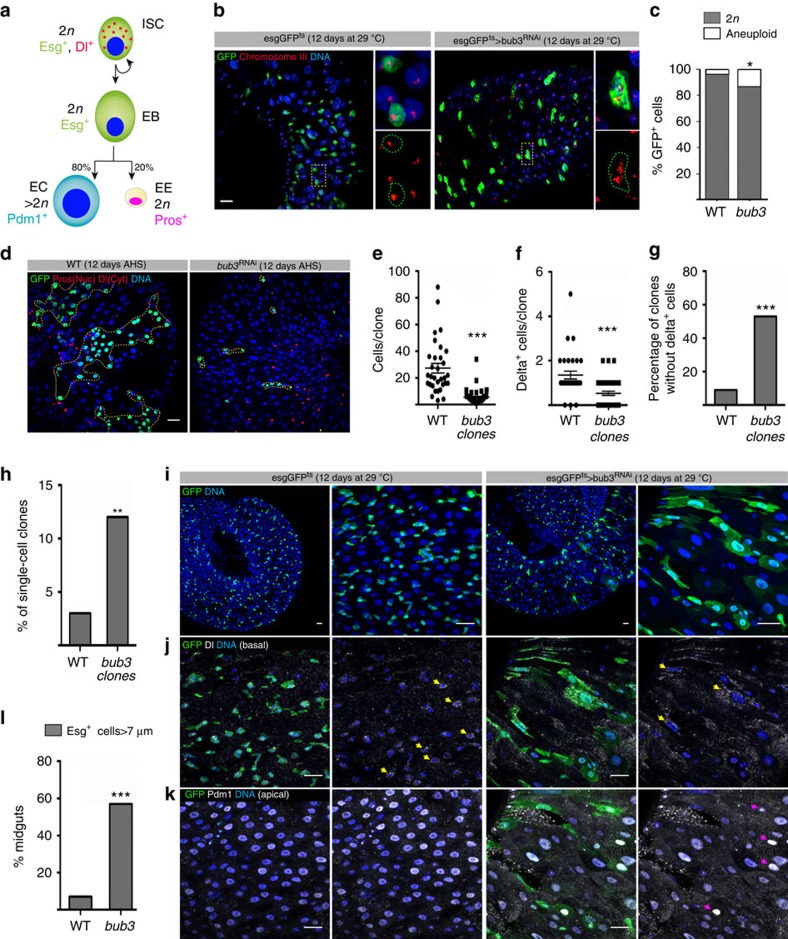
Intestine stem cells undergo premature differentiation upon *bub3* depletion. (**a**) Intestinal stem cell (ISC)s division scheme: ISC expresses Escargot (Esg) and Delta (Dl), divides asymmetrically to self-renew and to generate an enteroblast (Esg^+^) that generate enterocytes (ECs) (Pdm1^+^) that undergo polyploidization and enteroendocrine cells (EEs), diploid (Pros^+^). (**b**) Fluorescent *in situ* hybridization in Esg^+^ (green) clones. Wild-type ECs appear homogeneous in size and the FISH probes appear clustered in a large dot. *bub3*^RNAi^ inset shows a nucleus with abnormal morphology, never seen in WT and with several unclustered signals that allow the distinction between ECs (>2 n, that would still be Esg^+^) and non-euploid Esg^+^ cells. Scale bar, 10 μm. (**c**) Fluorescent *in situ* hybridization quantification (*n*=99 and 246 WT and *bub3*^*RNAi*^ Esg^+^ cells, respectively). Statistical significance determined using Fisher's exact test (Fet). *(*P*=0.012). (**d**) WT or *bub3*^*RNAi*^ GFP+ MARCM clones 12 days after heat shock (AHS) (in yellow), Dl (cytoplasmic red) marks SCs and Pros (nuclear red) marks EEs. Scale bar, 20 μm. (**e**) Quantification of number of cells/clone in WT and *bub3*^RNAi^ (*n*=31 and 45 clones, respectively). The line represents the mean and the error bars the s.d. Statistical significance determined using an unpaired *t*-test. ***(*P*<0.0001). (**f**) Quantification of Delta^+^ cells in WT and *bub3*^RNAi^ (*n*=31 and 45 clones, respectively). The line represents the mean and the error bars the s.d. Statistical significance determined using unpaired *t*-test. ***(*P*<0.0001). (**g**) Percentage of WT and *bub3*^RNAi^ (*n*=31 and 45 clones, respectively) without Dl^+^ cells. Statistical significance determined using Fet ***(*P*<0.0001). (**h**) Percentage of single cell clones in WT and *bub3*^RNAi^ (*n*=32 and 55 clones, respectively). Statistical significance determined using Fet. **(*P*=0.029). (**i**) WT and *bub3*^RNAi^ midguts expressing Esg GFP (green). DNA is in blue. Scale bars, 20 μm. (**j**) WT or *bub3*^RNAi^ midguts basal side with Dl (labelling ISCs, in white) and GFP. Wild-type Esg^+^ cells remain basally located and some express vesicular Dl, ISC marker (yellow arrows). Scale bars, 20 μm. (**k**) Apical side of the same WT or *bub3*^RNAi^ midguts than in (**j**) with GFP and Pdm1. Esg^+^, Pdm1^+^ cells are only observed in *bub3*^RNAi^ midguts (magenta arrows). Scale bars, 20 μm. (**l**) Percentage of Esg^+^ cells with nuclear size >7 μm in WT (*n*=40) and *bub3*^RNAi^ (*n*=54) midguts. Statistical significance determined using Fet ***(*P*<0.0001).

We then analyse adult ISCs using the Mosaic analysis with a repressible cell marker (MARCM) technique to induce expression of *bub3*^RNAi^. ISCs divide asymmetrically to self-renew and to generate one enteroblast (EB). EBs differentiate either into enteroendocrine cells (EEs) or enterocytes (ECs) that undergo polyploidization (Fig. 6a). First, to ascertain that *bub3* RNAi triggers aneuploidy in ISCs, we used the temporal and regional gene expression targeting (TARGET) method (EsgGal4>GFP, tubGal80^ts^) to express *bub3*^RNAi^ in adult ISCs and progenitor cells by shifting the temperature during 12 consecutive days to degrade the Gal4 inhibitor, tubGAL80. FISH revealed that 14.2% of *bub3*^*RNAi*^ Esg^+^ cells were aneuploid with only 4.3% in WT (Fig. 6b,c).

Clonal analysis revealed a significant reduction of the size of *bub3*^RNA*i*^clones, which contained on average 5.4 cells per clone (*n*=45 clones from six guts), as compared with WT clones having 27 cells (*n*=31 clones from eight guts Fig. 6d,e). Furthermore, *bub3*^RNA*i*^ clones presented less ISCs (Delta-Dl^+^) per clone and even clones without ISCs (Fig. 6f,g). In addition, analysis of single cell clones resulting from MARCM labelling of differentiating daughter cells demonstrated that these were more abundant in *bub3*^RNA*i*^ conditions than WT (*bub3*=12.7% compared with WT=3.1%; Fig. 6h). Thus, *bub3*^RNA*i*^ limits the ability of ISCs to produce progeny, promotes ISC loss and results in more single differentiated cells.

To further assess the cause of reduced clone growth, we inactivated *bub3* in all Esg^+^ cells (ISCs and progenitor cells). In WT, EsgGAL4>GFP marks diploid ISCs and progenitor cells. Upon *bub3*^RNA*i*^ expression, large polyploid nuclei could be seen marked by GFP, which was particularly evident in the anterior-most part of the posterior midgut (Fig. 6i). Although only 7.5% of WT guts presented these cells, 57.4% of *esgGAL4*>*bub3*^RNAi^ guts showed this phenotype (Fig. 6l). Many of the large polyploid nuclei were positive for the differentiated enterocyte marker Pdm1 suggestive of ISC terminal differentiation (Fig. 6k). In addition, some of the large polyploid nuclei were positive for the ISC marker, Dl, suggestive of polyploidization of aneuploid ISCs (Fig. 6j).

Together these data suggest that *bub3* aneuploidy in ISCs leads to a reduction in the ability of ISCs to produce progeny, which is at least in part due to ISC differentiation and polyploidization.

### *SakOE,mad2* brains display decreased tumourigenic potential

**Figure 7:**
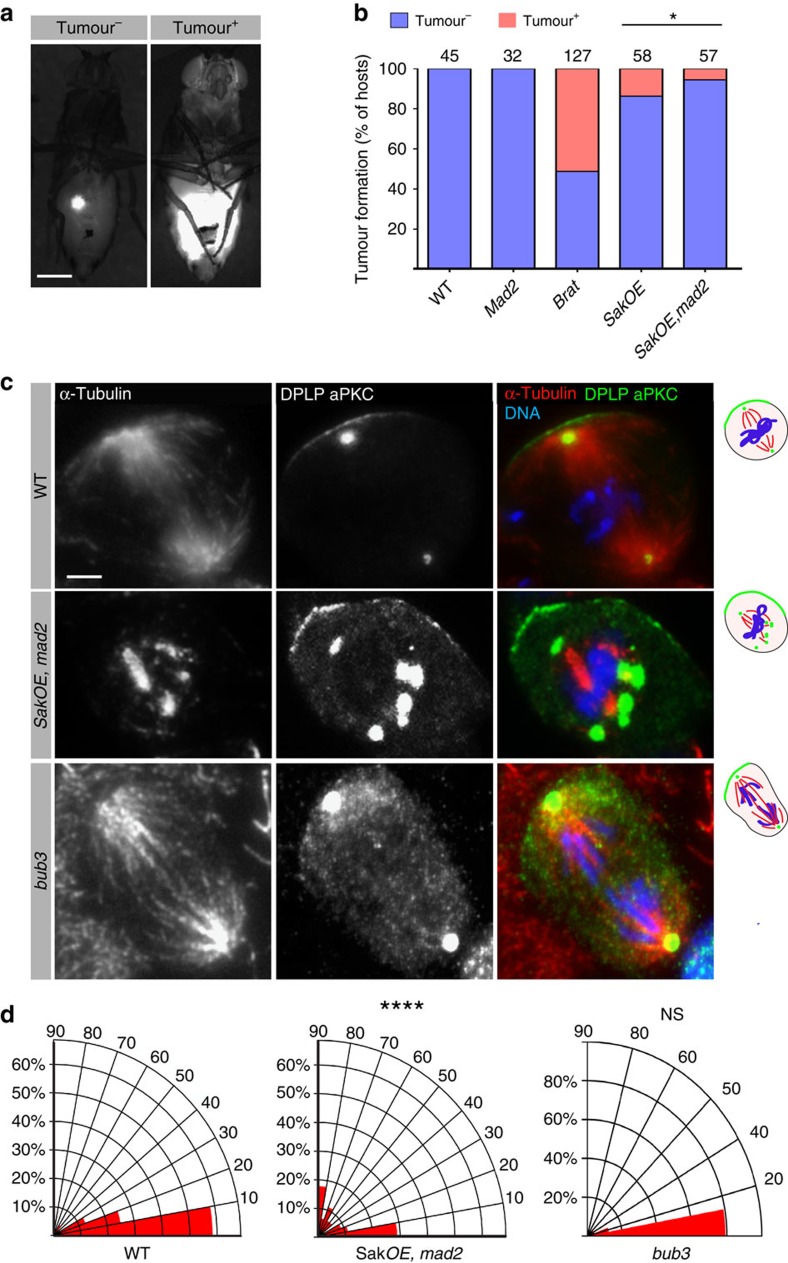
Aneuploid brains have reduced tumorigenic capacity. (**a**). Pictures of WT adult host flies transplanted with Tubulin-GFP (left) or Tubulin-GFP; *SakOE,mad2* (right) pieces of L3 brains. Scale bars, 400 μm. (**b**) Graph bars showing the quantification of tumour formation for indicated genotypes. Numbers on top of each column indicate the number of transplants performed. Statistical significance was determined using Fet *(*P*=0.03). (**c**) Immunostaining of WT, *SakOE,mad2* and *bub3* L3 Nbs with α-tubulin (left and in red in the merged panel), dPLP (*Drosophila* pericentrin-like protein) and aPKC (middle panel, shown in green in the merged panel) antibodies. DNA is shown in blue. Scale bar, 2 μm. (**d**) Quantification of mitotic spindle orientation at anaphase in WT (*n*=24), Sak*OE*, *mad2* (*n*=45) and *bub3* (*n*=24). Only Sak*OE*,*mad2* Nbs present spindle position defects. Statistical significance was determined using unpaired *t*-test ****(*P*<0.0001).

Aneuploidy can be seen as tumour suppressor or oncogenic^16^,^56^. *SakOE* brains induced tumour formation, due to spindle positioning defects, which resulted in the expansion of the Nbs pool^12^. We wondered whether the addition of aneuploidy to a tumour-permissive condition would influence the tumourigenic capacity of *SakOE* Nbs by transplanting *SakOE,mad2* brain pieces into the abdomen of WT hosts. Interestingly, we found a clear decrease in the tumourigenic potential of *SakOE,mad2* brains (Fig. 7a,b). Nevertheless, these brains were still tumourigenic, while other aneuploid mutants such as *bub3* (ref. 11) were not.

Since brains that contain Nbs that display asymmetric cell division defects were found to be tumourigenic in transplantation assays^11^,^57^, we reasoned that *SakOE,mad2* Nbs might maintain the tumourigenic potential because of defects in spindle positioning and asymmetric cell division. We analysed and compared spindle positioning relative to the apical aPKC crescent in *SakOE,mad2 and bub3* brains. As expected, *SakOE,mad2* Nbs presented defects in spindle positioning in a significant (using a student *T*-test) proportion of cells, while in *bub3* Nbs, which contained always two centrosomes, spindles were correctly positioned (Fig. 7c,d).

Together, we conclude that in the fly brain, aneuploidy decreases NSCs tumourigenic potential.

## Discussion

Maintenance of an euploid genome in the stem cell compartment is essential to control stem cell self-renewal and to produce genetically stable daughter cells. Cells and organisms have therefore developed surveillance mechanisms that detect the presence of abnormal karyotypes that are normally eliminated by apoptosis^13^,^14^,^33^. In this study we have found a novel response of *Drosophila* NSCs or ISCs to aneuploidy, which prevent cells with abnormal genomes from cycling. We show that these cells undergo premature differentiation. Importantly, this response is not exclusive to *Drosophila*. Premature differentiation of NSCs was observed in the mouse brain, but only when apoptosis was inhibited^7^. Thus, while in *Drosophila* premature differentiation represents a primary response, in the mouse brain this might represent a backup mechanism when the mechanisms that regulate cell death are not efficient.

One challenge in the future will be to identify the sensor(s) and effector(s) present in NSCs and ISCs that influences the shift towards differentiation. Several studies have recently shown that cell cycle timing dictates cell fate decisions. A functional organ of the correct size requires a tight temporal control of cell cycle progression and differentiation. Reduced proliferation and cell death in the CNS leads to the formation of microcephalic brains^7^,^58^ (and our results), whereas over proliferation and/or lack of differentiation might lead to tumour formation. Neurogenic divisions of neural progenitors are accompanied by G1 lengthening prior to differentiation in the developing mouse^47^ and Xenopus^59^ nervous systems. This is also the case in both *SakOE,mad2* and *bub3* mutant NSCs that presented a significant delay in the G1 phase, due to aneuploidy.

Aneuploidy has been proposed to be at the origin of a general stress response in cells, mediated by p53-dependent mechanisms. Moreover, extension of G1 due to prolonged prometaphase arrest p38 dependent has also been implicated in eliminating proliferative cells containing spindle abnormalities^60^. Nonetheless, we ruled out the possibility that aneuploid fly NSCs undergo a p38-mediated arrest, as lowering p38 levels did not restore proliferation of aneuploid NSCs. Moreover, even if p53 was upregulated in aneuploid brains, Nbs did not show frequently p53 increased levels. Instead other brain cell types, such as GMCs, neurons and other cell types, probably glia, showed an increase in p53. It is thus possible that p53 upregulation in the brain reflects a stress response that unlike in other cell types, does not culminate with cell death by apoptosis.

Therefore other mechanisms induced during or in response to G1 lengthening seem to be responsible for premature differentiation in fly Nbs. A likely possibility is that during this abnormal G1 lengthening the transcription factor Pros, which promotes differentiation and inhibits proliferation, translocates to the nucleus to promote differentiation. In agreement, Pros co-localizes with Dpn in Nbs from aneuploid brains and reduction of Pros levels results in increased Nb number. Through binding to its adapter Mira, Pros is targeted to the basal cortex and inherited by the GMC and upon separation from Mira, Pros translocates to the GMC nucleus^9^,^53^. Importantly, Mira degradation is not required for Pros release^53^. Therefore, it is possible that an extended G1 phase allows for Pros release, which would be sufficient to trigger differentiation of Nbs.

Our work extends the list of adverse situations to aneuploidy and might reflect a common response of stem cells to hazardous situations to inhibit the proliferation of damaged genomes.

## Methods

### Fly stocks and genetics

Flies were raised on cornmeal medium at 22 or 25 °C. We used the following stocks Tubulin-RFP^12^ and Tubulin-GFP^12^, *SakOE*^12^, *bubR1*^*ΔKEN*^*, bubRI*^25^, *hsflp*; Tub>Gal80>Gal4, UAS-mCD8-RFP (provided by Yohanns Bellaiche), *worGAL4* (provided by Chris Doe), *brat*^*06028*^, Histone 2B-RFP^57^ (Bloomington (Bl) #23650), *Act-Gal4 (*Bl#25374*), Eng-Gal4, UAS-RFP* (Bl#30557), UAS-p35 (Bl#5072/73), *mad2* (Bl#22495), *bub3*^30^, *bub3*^RNAi^ (Bl#32989) *Prospero*^RNA*i*^ (Bl#26745), pros^17^ (Bl#5458), FUCCI#21 (Bl#55117)^50^, p53^RNAi^ (Bl#41638), p53^R155H^ (Bl#8419), esgGAL4 tubGAL80ts UAS-GFPhsFlp tubGAL4 UAS-nlSGFP (provided by Bruce Edgar), FRT 40A tubGAL80 (MARCM 40A)^61^, p53 reporters (p53-GFP-NLS and p53-GFP-cytoplasmic^39^), *dacapo*^RNAi^ (Bl#36720), *p38*^RNAi^ (Bl#35252) and *w118* and *wf* were used as control strains. We used either *SakOE,mad2* homozygotes or *SakOE,mad2/mad2* that gave similar phenotypes. Overexpression of FUCCI markers, p35, p53^R155H^ and depletion of *Prospero, bub3, dacapo, p38* and *p53* were carried out using the GAL4/UAS system^62^. We used the worGAL4 driver for expression in Nbs, engGAL4 driver for expression in the disc and ActGAL4 for ubiquitous expression.

Clonal expression of bub3^RNAi^ was achieved using a flip-out strategy^41^, using the Tub-FRT-GAL80-FRT-GAL4 transgene. Clones were induced on second-instar larvae by heat shock (40 min 37 °C) and mid third-instar larval brains were analysed. In the analysis of intestines, only female flies were analysed. MARCM clones were generated as previously described^63^. To measure cell cycle parameters in *p38*^RNAi^ and *dacapo (p27)*^RNAi^ conditions, recombinants lines *mad2,p38*^RNAi^ and *mad2,dacapo*^RNAi^ were crossed with *worGAL4; SakOE,mad2* lines. *Cyo; SakOE,mad2/mad2* larvae were used as controls. Adult flies were kept at 25 °C (unless otherwise noted) in freshly yeasted tubes, changed every 2–3 days. Crosses were raised at 25 °C and 3–5-days-old adults were heat shocked for 35 min at 36.5 °C. For temperature shift experiments crosses were set up and maintained at 18 °C until adulthood. Adult flies (3–5 days old) were shifted to 29 °C for 12 days.

### Staging fly cultures

Six hours egg collection were performed at for each genotype. Vials were kept at 25 °C until larvae were reaching the required developmental stage (early, mid or late third instar at 72, 96 or 120 h after egg deposition).

### Immunohistochemistry and fixed tissue imaging

For immunohistochemistry, brains from third instar larvae were dissected in PBS, fixed for 30 min in 4% paraformaldehyde in PBS or in PBS with 0.1% Triton X-100; They were washed three times in PBS-T (PBS, 0.3% Triton X-100) and incubated O/*N* at 4 °C with primary antibodies diluted in PBS-T. After washing in PBS-T three times, brains were incubated O/*N* at 4 °C with secondary antibodies and Hoechst (0.5 μg ml^−1^ in PBS-T), washed once more in PBS and mounted in mounting media (1.25% *n*-Propyl Gallate, 75% glycerol, 25% H_2_0).

Midgut fixation and immunofluorescence staining were performed as described previously^61^. Briefly adult female intestines were dissected in PBS and fixed for 2 h in 4% paraformaldehyde. Intestines were rinsed in PBT (PBS containing 0.1% Triton X-100), trimmed and incubated for at least 30 min in PBS containing 50% glycerol, then equilibrated in PBT to osmotically clean the lumen before antibody incubations.

Primary antibodies used: guinea pig anti-Deadpan (Dpn) (1:1,000, gift from J. Skeath); Rabbit (Rb) anti Dpn (raised by expressing a fusion protein provided by J. Skeath) rat anti-Elav (1:100, 7E8A10, DSHB); mouse anti-Prospero (1:500, MR1A-c, DSHB), Rb anti-GFP (1:400, A11122, Molecular Probes, Invitrogen); rat anti-RFP (1:200, 5F8, ChromoTek); mouse anti-α-tubulin (DM1Α) (1:500, Sigma-Aldrich); Rb anti-Caspase-3 (1:75, Asp175, Cell Signalling); mouse anti-Miranda (1:20, gift from F. Matzuzaki); Rb anti-Miranda (1:200, a gift from the Yan lab); Rb anti H2Av pS137 (1:500, TEBU-bio); Rb anti PKC (1:100, sc-216, Santa Cruz); Rb anti-dPlp (1:500) and guinea pig anti-Cnn (1:1000, both a gift from the Raff lab) (1:,1000); Rb anti phospho histone3 pSer^10^ (1:500, Sigma); mouse anti- Delta ECD C594.9B (ascites, 1:2,000, (DSHB); chicken anti-GFP (1:4,000, Abcam); Rb anti-Pdm1 (1/1,000, a gift from X. Yang); mouse anti-mono and polyubiquitinylated conjugates (1:100, FK2, Enzo Life Science); and mouse anti-ATP5a (1:100, ab14748, Abcam). Secondary antibodies used: fluorescent conjugated Alexa Fluor 488, Alexa Fluor 546 and Alexa Fluor 647 (Molecular probes, Invitrogen). For actin staining we used Alexa Fluor 488 (1:250), 568 (1:100) and 647 (1:80) Phalloidin (Molecular probes, Invitrogen). All images were acquired on a Nikon A1R inverted TiE microscope with a 40 × 1.3 NA or 60 × 1.4 NA objectives in NIS Element software or an Inverted Laser Scanning Confocal Leica SP8 MP-FLIM/FCS with 40 × , NA 1.3 objective. Interval for *z*-stacks acquisitions was set up from 0.2 μm (FISH, spindle orientation) to 1 μm (whole mount brains). Images were processed with Fiji and Adobe Photoshop.

### Live imaging

Mid third-instar larval brains were dissected in Schneider's *Drosophila* Medium (21720-024, Gibco) supplemented with 10% heat-inactivated fetal bovine serum (10500, Gibco), Penicillin (100 units ml^−1^) and Streptomycin (100 μg ml^−1^) (Penicillin-Streptomycin 15140, Gibco). Two to four brains were placed on a glass bottom 35 mm dish (P35G-1.5-14-C, MatTek Corporation) with 10 μl of medium, covered with a permeable membrane (Standard membrane kit, YSI) and sealed around the membrane borders with oil 10S Voltalef (VWR BDH Prolabo). Images were recorded using a Yokagawa CSU-X1 spinning head mounted on a Nikon TiE inverted microscope. The microscope was equipped with an EMCCD Evolve 512 × 512 (Photometrics) and controlled by the Metamorph software 7.7 (Molecular devices). Four-dimensional *z*-stacks of 10-30 μm at 1 μm intervals were acquired every 30 or 60 s using a 60 × NA 1.4 oil immersion objective. Images were processed with ImageJ.

### EdU (5-ethynyl-2'-deoxyuridine) incorporation

Mid third-instar larval brains were dissected in Schneider's *Drosophila* Medium used for live imaging and incubated for 2 h at 25 °C in the same medium implemented with 100 μM EdU (C10338, Invitrogen). Brains were then washed in PBS, fixed and immunostained. EdU detection was realized after the secondary antibody detection, according to the manufacturer instructions.

### DNA damage assay

Mid third instar larval brains were dissected in Schneider's *Drosophila* Medium used for live imaging and incubated for 30 min at 25 °C in medium implemented with 100 μg ml^−1^ of aphidicolin (A0781, Sigma-Aldrich). Brains were washed three times in PBS and incubated 1 h in medium without drug. Brains were fixed and stained as described above.

### Reactive oxygen species detection

Mid third instar larval brains were dissected in PBS supplemented with dihydroethidium (final concentration 30 μM, D-1168, Life Technologies) for 10 min, washed two times in PBS (5 min per wash) and immediately mounted in a glass slide for live imaging.

### Fluorescent *in situ* hybridization

Oligonucleotides probes for AACAC repeats (chromosome II) and dodeca sequence (Chromosome III)^64^,^65^ were synthesized with a 5′CY3 and FAM488 fluorescent dye, respectively. We used the following sequences: 5' CY3-AACACAACACAACACAACACAACACAACACAACAC, FAM488-CCCGTACTGGTCCCGTACTGGTCCCG. Our FISH protocol was adapted from previously described methods^64^,^65^ (with the exception that all the steps were done on whole brains in the PCR machine instead of glass slides). Fluorescent *in situ* hybridization in the brains was performed by dissecting third-instar larval brains in PBS and fixed 30 min in 4% formaldehyde in PBS with 0.1% tween 20 (162312, Panreac Sintesis). For the intestine, the identification of esgGFP^+^ cells is required. Intestines were dissected and fixed for 40 mn in PFA 4%, washed 3 times in PBS-T (PBS, 0.3% triton) and incubated with an anti-GFP antibody (1:250) overnight (O/*N*) washed three times in PBS-T and incubated with secondary antibody O/*N* at 4 °C. Intestines were then washed three times in PBS-T and fixed another time for 20 mn in PFA 4% before proceeding with the probe hybridization. Brains or intestines were then washed three times in PBS, once in 2xSSCT (2XSSC (EU0300, Euromedex)/0.1% tween-20), once in 2XSSCT/50% formamide (47671, Sigma). For the pre-hybridization step, brains were transferred to a PCR tube containing 92 °C pre-warmed 2XSSCT/50% formamide and denaturated 3 min at 92 °C. Brains were then hybridized 5 min at 92 °C with previously denaturated DNA probe (40-80 ng) in hybridization buffer (20% dextran sulfate (D8906, sigma)/2XSSCT/50% deionized formamide (F9037, Sigma), 0.5 mg ml^−1^ salmon sperm DNA (D1626, Sigma)). 3 min after denaturation at 92 °C, tubes were left O/*N* at 37 °C. Samples were then washed with 60 °C pre-warmed 2XSSCT for 10 min, and one time 5 min in 2XSSCT at room temperature. Samples were then stained with DAPI (in 2XSSC) for at least 30 min and mounted using our standard mounting medium (1.25% n-propyl gallate (Sigma, P3130), 75% glycerol (bidistilled, 99.5%, VWR, 24388-295), 25% H_2_O).

FISH signals were quantified by manual scoring of the individual slices for each *z*-stack.

### Image analysis quantification and statistical analysis

All analysis or measurements were performed on Fiji or ImageJ software. Quantifications were performed manually using the cell counter tool. Brain areas were measured by ImageJ. In the quantifications of Dpn^+^, Pros^+^ and Elav^+^ cells (Figs 3 and 5), only nuclear Dpn, Pros and Elav signals were considered as scoring positive. To quantify type-I Nbs, only Dpn^+^ cells displaying nuclear sizes >8 μm were considered. Quantifications were always done on the same area of brains (ventral side) using 3 Z (steps of 1 μm) around the plane were the maximum of Nbs are present. For CC3 quantification, the whole CB region was analysed.

In Fig. 6, only posterior midgut tissue was analysed and only clones of minimum two GFP-positive cells were scored and at least six different midguts of each genotype were analysed. The presence of Esg^+^ cells with nuclear size above 7 μm was only observed and scored in the distal midgut loop previously reported as highly proliferative and being a dynamic part of the tissue^66^. Statistical analysis was performed with Graph Pad Prism using the tests mentioned in the figure legends.

### Analysis of spindle positioning

Analysis of spindle position relative to the apical aPKC crescent position was performed by staining fixed brains with anti-aPKC, anti-α-tubulin and anti-Cnn antibodies. For *SakOE,mad2* Nbs analysis, we only scored mitotic cells with more than two centrosomes and where an aPKC crescent could be clearly distinguished. The angle between the spindle axis and the aPKC crescents was determined at early anaphase to avoid any bias in possible rotation during early mitotic stages, using the measurement tool in ImageJ. The data were analysed for statistical significance using the two-tailed *t*-test function in Prism.

### Western blots

For each genotype, 15 brains from third-instar larvae were dissected in PBS, 1% protease inhibitor cocktail and extracted in 100 μl of cold protein extract buffer (50 mM trisHCl pH 7.4 (200923A, Euromedex), 50 mM NaCl (27810, Prolabo), 1 mM EDTA (E9884, Sigma), 0.25% DOC (D6750, Sigma), 0.1% SDS (EU0660, Euromedex), 1 mM PMSF (P7626, Sigma), 1‰, DTT (D0632), 1% of protease inhibitor solution (1 mM benzamidine-HCl (B6506, Sigma), 0,1 mg ml^−1^ phenanthroline (131377, Sigma), 1 mg ml^−1^ aprotinin (A6103, Sigma), 1 mg ml^−1^ leupeptin (L2884, Sigma), 1 mg ml^−1^ pepstatin A (P5318, Sigma)). Protein extracts were separated by SDS–polyacrylamide gel electrophoresis with either a 10% BIS-TRIS or a 3-8% TRIS-Glycine NuPAGE gel (Invitrogen) and then transferred on a nitrocellulose membrane (Protran BA83, Whatman). Membranes were probed with antibodies anti-Fzy (1:1,000, a gift from J. Raff lab) and anti-α-tubulin (DM1Α) (1:1,000, Sigma-Aldrich).

### Transplantation assays

The injection protocol was adapted from previously described method^57^. Mid third-instar larval brains expressing tubulin-GFP in either WT or mutant background were dissected in PBS, rinsed three times, sliced in several pieces and transplanted into adult host females abdomen using a elongated Pasteur pipette (drawn out in a flame to a tip diameter of approximately 100 μm) connected to a hand-driven microinjector. After injection, flies were transferred to 18 °C for 24 h and then to 25 °C in tubes with males. Five days after transplantation, flies were scored on a daily basis. Hosts were scored positive for tumour formation when a high GFP intensity signal could be easily detected in a homogeneous way within the abdomen. This normally occurs, independently of the genotypes tested in the study, between days 10 and 15. Injected flies were monitored until day 25 and we have never observed late onset tumours.

## Additional information

**How to cite this article:** Gogendeau, D. *et al.* Aneuploidy causes premature differentiation of neural and intestinal stem cells. *Nat. Commun.* 6:8894 doi: 10.1038/ncomms9894 (2015).

## Supplementary Material

Supplementary Information Supplementary Figures 1-10 and Supplementary Tables 1-2

Supplementary Movie 1 WT Neuroblast expressing a H2b-RFP (shown in red) transgene to visualize chromosomes and a a-tubulin-GFP transgene (shown in green) to visualize the mitotic spindle.

Supplementary Movie 2 SakOE (video 2)Neuroblast expressing a H2b-RFP (shown in red) transgene to visualize chromosomes and a a-tubulin-GFP transgene (shown in green) to visualize the mitotic spindle.

Supplementary Movie 3 SakOE,mad2 Neuroblast expressing a H2b-RFP (shown in red) transgene to visualize chromosomes and a a-tubulin-GFP transgene (shown in green) to visualize the mitotic spindle and undergoing multipolar division.

Supplementary Movie 4 SakOE,mad2 Neuroblast expressing a H2b-RFP (shown in red) transgene to visualize chromosomes and a a-tubulin-GFP transgene (shown in green) to visualize the mitotic spindle.This cell present a bipolar division with lagging chromosomes.

Supplementary Movie 5 SakOE,mad2 Neuroblast expressing a H2b-RFP (shown in red) transgene to visualize chromosomes and a a-tubulin-GFP transgene (shown in green) to visualize the mitotic spindle.Cell presenting a cytokinesis defect

Supplementary Movie 6 bubR1*,Sak Neuroblast expressing a H2b-RFP (shown in red) transgene to visualize chromosomes and a a-tubulin-GFP transgene (shown in green) to visualize the mitotic spindle.This cell present a bipolar division with lagging chromosomes.
