# Supplementary material for: Aneuploidy causes premature differentiation of neural and intestinal stem cells
Source: Nat Commun. 2015 Nov 17;6:8894. doi: 10.1038/ncomms9894 (PMC4660207; doi:10.1038/ncomms9894)
Supplement: Supplementary Information — Supplementary Figures 1-10 and Supplementary Tables 1-2 [file ncomms9894-s1.pdf]

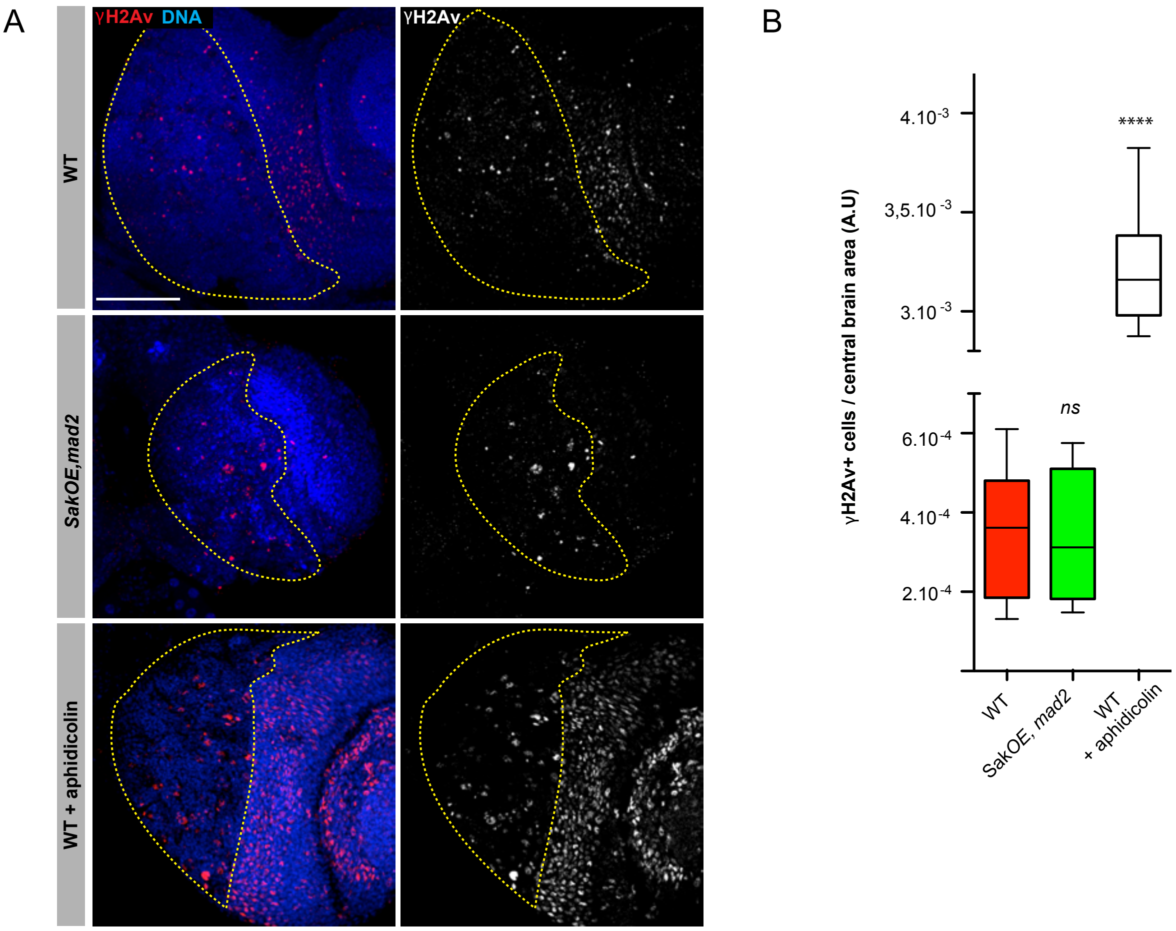

Supplementary Fig. 1- Aneuploid brains do not show increased DNA damage

(A) Pictures of Wild Type (WT) (top), *SakOE,mad2* (middle) and WT incubated with aphidicolin (bottom) brain lobes stained with γH2Av antibodies (right and shown in red in the merged panel) to label sites of DNA damage. DNA is shown in blue. The dashed line marks the central brain region. Scale bar= 100 µm.

(B) Column chart showing the quantification of the ratio between γH2Av positive cells per central brain area in WT (n=14 lobes, red column), *SakOE,mad2* (n=9 lobes, green column) and WT brains incubated with aphidicolin (n=5 lobes, white column). The line represents the mean and the error bars the SD. Statistical significance was assessed was assessed by an unpaired t test. ns=non significant, **** ($p<0,0001$).

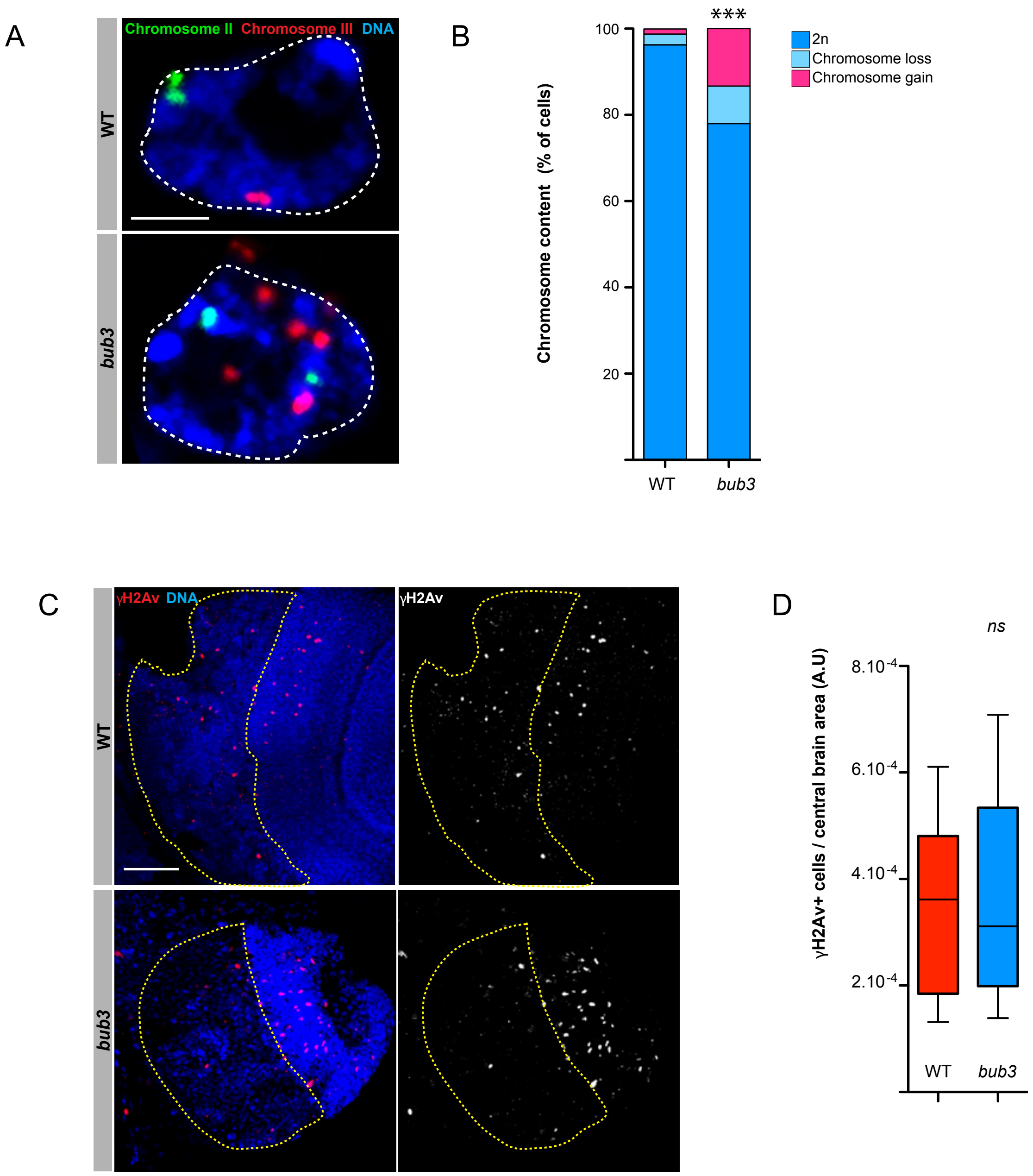

Supplementary Fig. 2- Quantification of aneuploidy levels in *bub3* neuroblasts
(A) *Fluorescent in situ hybridization* (FISH) staining using probes for chromosomes II (shown in green) and III (showed in red) in Wild type (WT, top) and *bub3* Nbs (bottom). Scale bar = 3,5 μm.
(B) Graph bar showing the quantification of chromosome content in WT (n=227 cells), and *bub3* (n=206 cells) Nbs. Statistical significance was determined using Fisher's exact test. *** (p<0,0001),
(A) Pictures of WT and *bub3* brain lobes stained with γH2Av antibodies to label DNA damage (right and shown in red in the merged panel). DNA is shown in blue. The dashed line marks the central brain region. Scale bar = 50 μm
(B) Column chart showing the quantification the ratio between γH2Av positive cells per central brain area in WT (n=14 lobes, red column) and *bub3* (n=13 lobes, blue column). The line represents the mean and the error bars the SD. Statistical significance was assessed by an unpaired t test: ns=non significant.

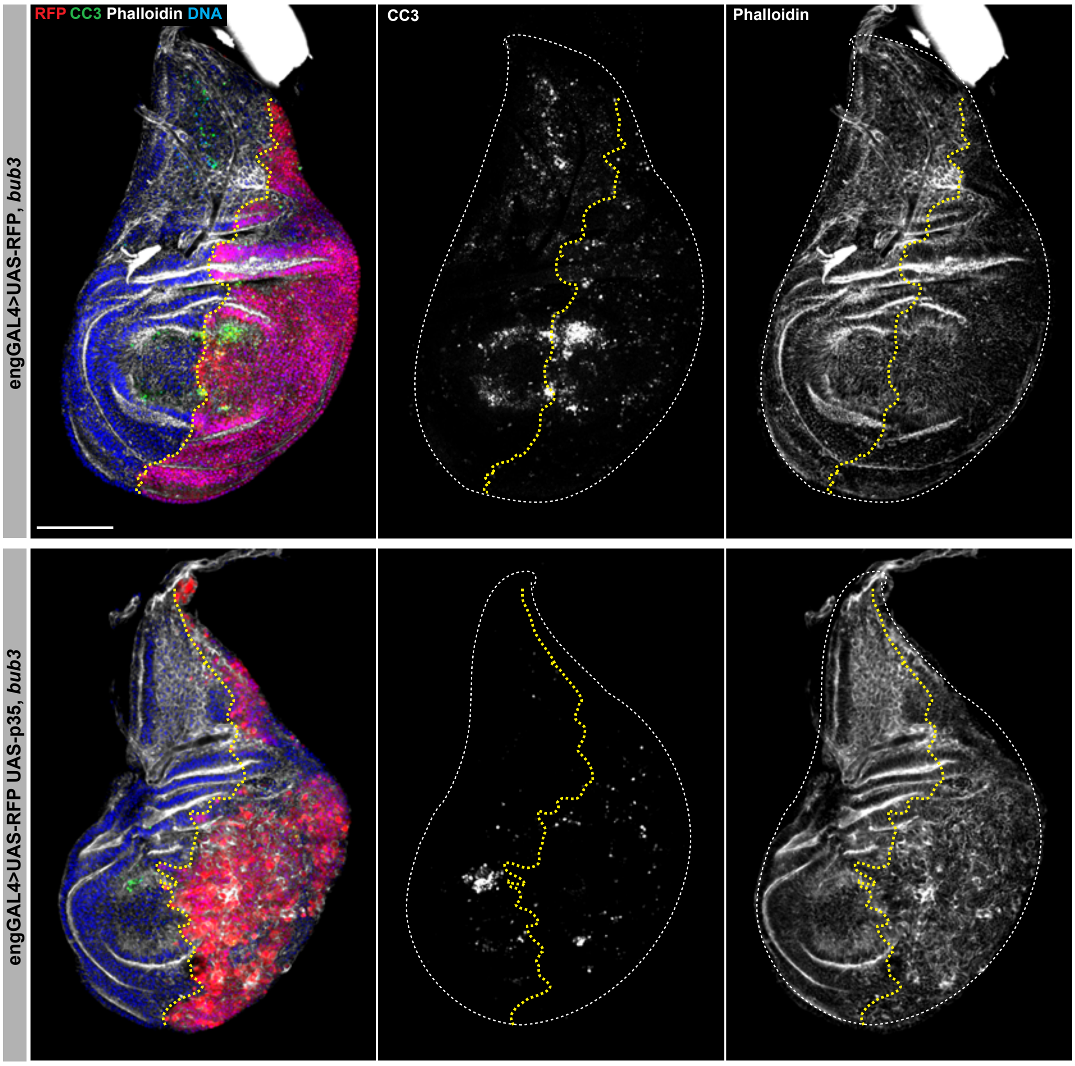

Supplementary Fig. 3- p35 expression inhibits apoptosis in *bub3* wing discs

Pictures of *bub3* mutant (top) and *bub3* mutant over-expressing the apoptosis inhibitor p35 (bottom) wing discs under the control of the eng-Gal4 promoter, which drives expression in the posterior compartment labeled by the yellow dashed line. RFP expression labels the eng-Gal4 compartment. Cleaved caspase 3 (CC3) (green, middle panel) marks apoptotic cells. Phalloidin (white, right panel) is used to show the overall morphology of the disc. DNA is shown in blue. Scale bar= 100 µm.

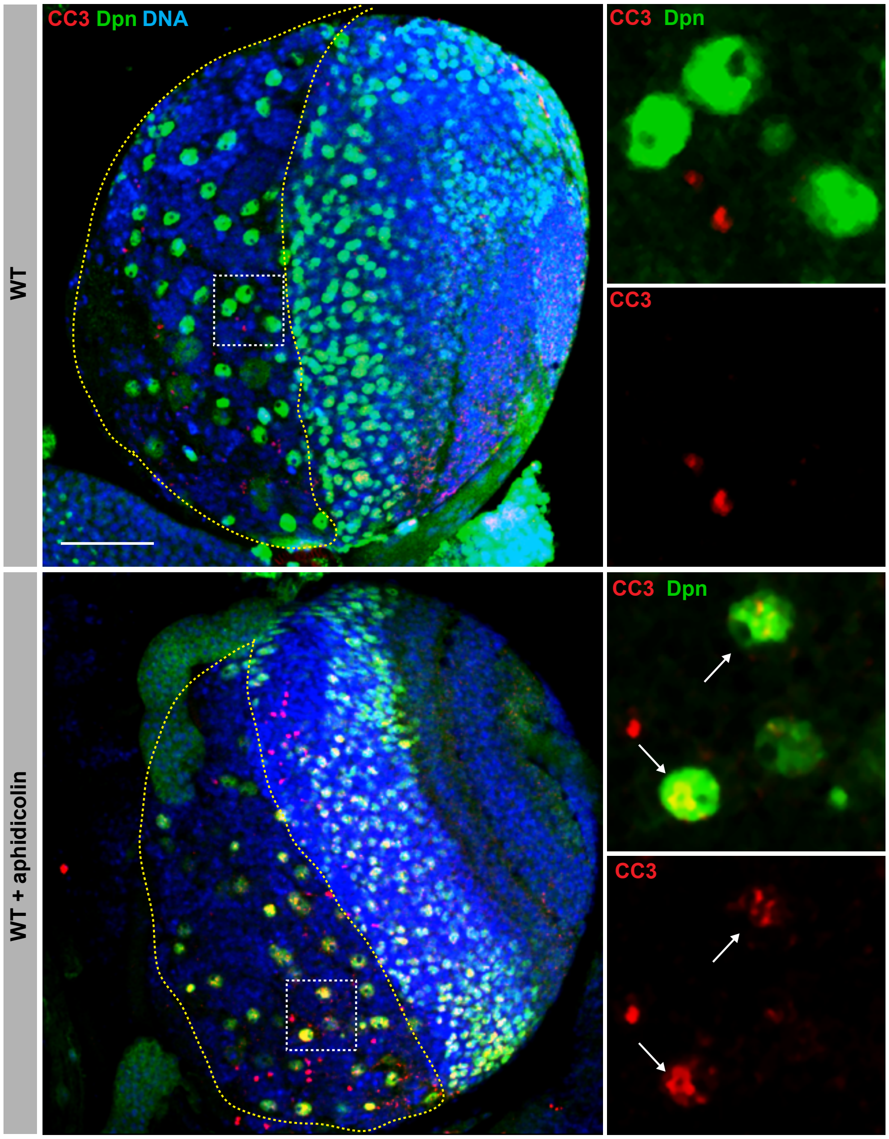

Supplementary Fig. 4- Apoptosis in brains with DNA damage
Wild type (WT, top) and WT aphidicolin incubated brains (bottom) stained with cleaved caspase 3 (CC3, red) to label apoptotic cells and Dpn to label Nbs (green), DNA is shown in blue. The yellow dashed line marks the central brain. Panels on the right are enlargements of the insets. $CC3^+$ Nbs are identified in aphidicolin treated brains (arrows). Scale bar= 50 µm.

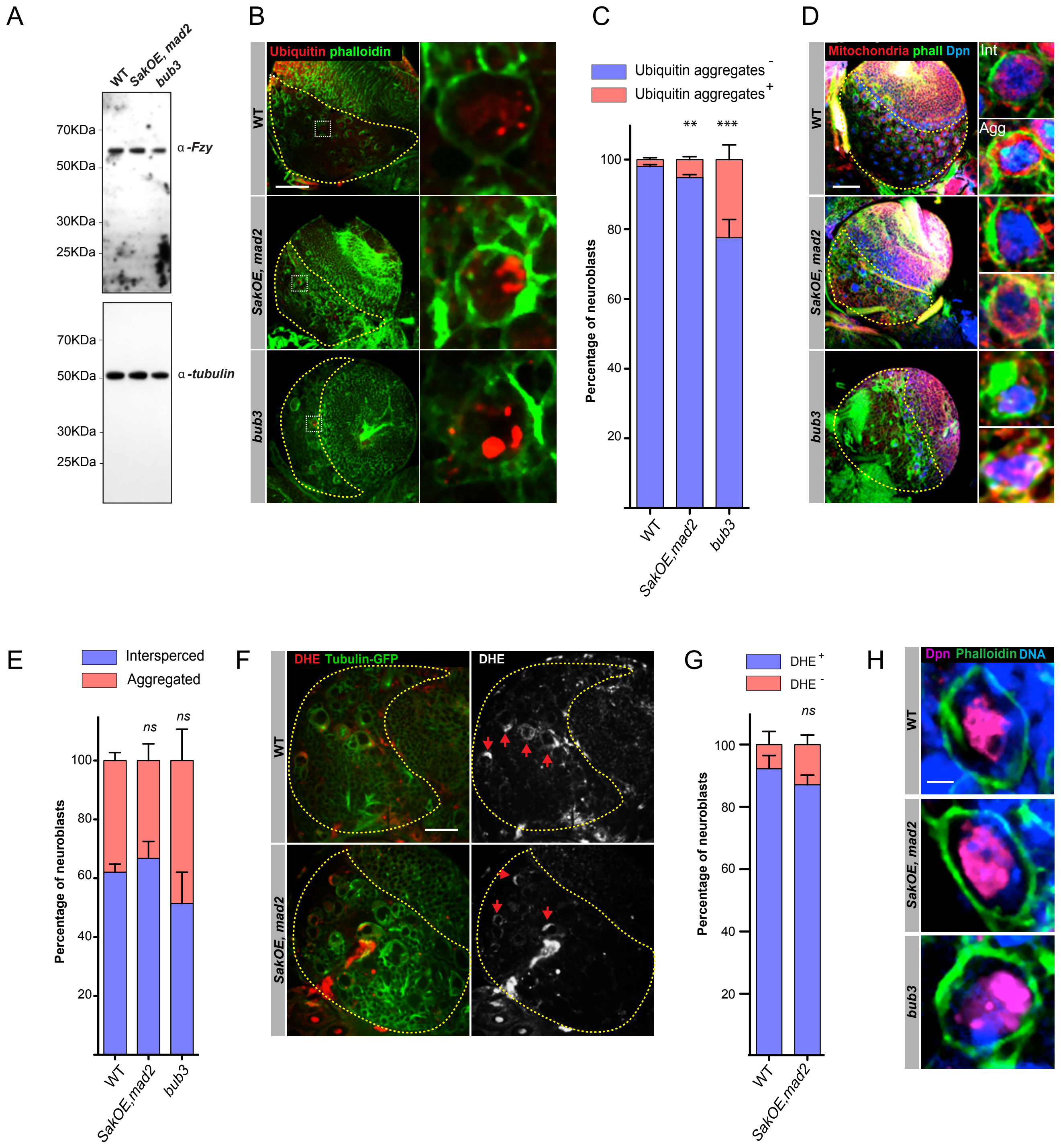

Supplementary Fig. 5- Stress responses in aneuploid brains

(A) Western blot of Wild type (WT), *SakOE,mad2* and *bub3* mutant brains extracts (15 brains) showing comparable levels of Fzy.

(B) Pictures of WT (top), *SakOE,mad2 (middle)* and *bub3 (bottom)* mutant brain lobes stained with FK2 antibodies to reveal ubiquitin aggregates (red) and Phalloidin (green). The panels on the right show enlargements of the insets. The yellow dashed line marks the central brain. Scale bar= 50 µm.

(C) Graph bars showing the quantification of Ubiquitin aggregates $^{+}$ Nbs in WT (n=325 Nbs from 7 brain lobes), *SakOEmad2* (n=279 Nbs from 11 brain lobes), and *bub3* brains (n=71 Nbs from 10 brain lobes). Statistical significance was determined using unpaired t-test. ** (p=0.0073) and ***(p=0.0002).

(D) Pictures of WT (top), *SakOE,mad2* (middle) and *bub3* (bottom) mutant brain lobes stained with antibodies that recognize mitochondria (red) and labeled with Phalloidin and (green). DNA is shown in blue. The panels on the right show enlargements of the insets. The yellow dashed line marks the central brain. Scale bar=50 µm. The insets show mitochondria morphology: intersperced (Int) or aggregated (Agg).

(E) Graph bars showing the quantification of mitochondria morphology in Nbs of WT (n=189 Nbs from 11 brain lobes), *SakOEmad2* (n=96 Nbs from 9 brain lobes), and *bub3* brains (n=38 Nbs from 7 brain lobes) Nbs. Statistical significance was determined using unpaired t-test. ns=not significant.

(F) Pictures of WT (top) and *SakOE,mad2* (bottom) mutant brain lobes expressing Tubulin-GFP (green) and incubated with Dihydroethidium (DHE) to reveal ROS production (red and right hand panel). Scale bar=50 µm.

(G) Graph bars showing the quantification of DHE^+ Nbs in WT (n=128 Nbs from 5 brain lobes), and *SakOE,mad2* (n=174 Nbs from 8 brain lobes) Nbs. Statistical significance was determined using unpaired t-test. ns=not significant.

(H) Pictures of WT, *SakOE,mad2* and *bub3* Nbs labeled with Dpn (purple), Phalloidin (green) and DNA in blue, to show intact membranes. Scale bar= 2.5 µm.

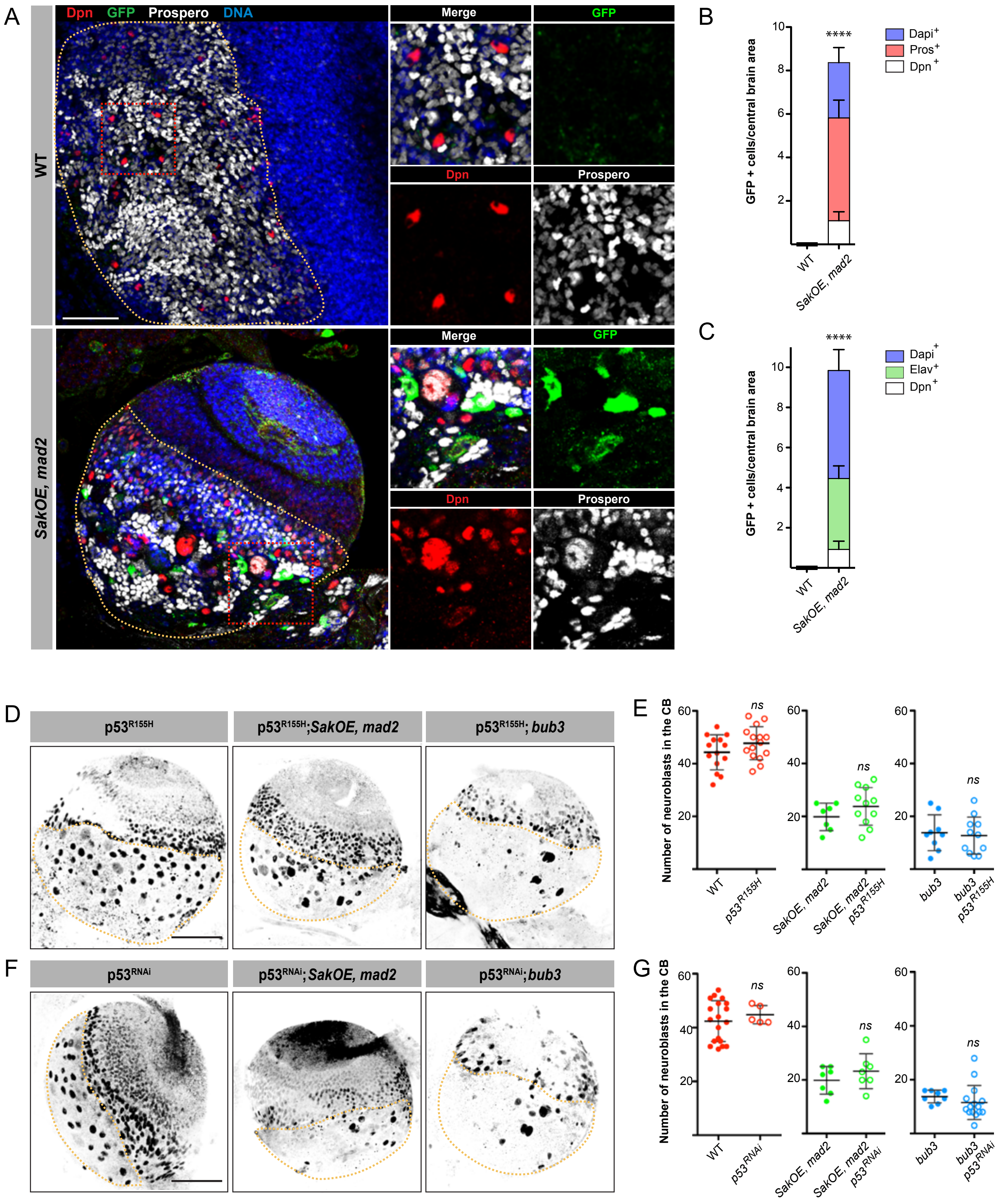

Supplementary Fig. 6- p53 in aneuploid brains

(A) Pictures of Wild type (WT, top) and *SakOE,mad2* (bottom) brain lobes expressing the GFP-p53 reporter (green), and labeled with Dpn (red, to show Nbs) and Pros (white, to show GMCs). Panels on the right show enlargements of the inset. The yellow dashed line labels the central brain. Scale bar= 50 µm.

(B) Graph bars showing the distribution of GFP^{+} cells per brain lobe in WT (n=17 brain lobes) and *SakOE,mad2* (n=11 brain lobes) brains taking into account Dpn and Pros to label Nbs and GMCs respectively. Statistical significance was determined using unpaired t-test. **** (p < 0,0001).

(C) Graph bars showing the distribution of GFP^{+} cells per brain lobe in WT (n=10 brain lobes) and *SakOE,mad2* (n=13 brain lobes) brains taking into account Dpn and Elav to label Nbs and neurons respectively. Statistical significance was determined using unpaired t-test. ****(p < 0,0001).

(D) Pictures of the expression of UAS-$p53^{R115H}$ in WT, *SakOE,mad2* and *bub3* mutant brain contexts (using a Nb specific Worniu-Gal4 driver) labeled with Dpn to visualize the Nbs. The yellow dashed line labels the central brain. Scale bar= 80 µm.

(E) Dot plot charts depicting the number of Nbs in the central brain (CB) area of UAS-$P53^{R115H}$ in WT (red circles, n=15 lobes), in *SakOE,mad2* (green circles, n=11 lobes), and *bub3 (*blue circles, n=11 lobes). Each dot corresponds to one brain lobe. The line represents the mean and the error bars the SD. Statistical significance was determined using unpaired t-test. ns=not significant.

(F) Pictures of the expression of $p53^{RNAi}$ (bottom) in WT, *SakOE,mad2* and *bub3* mutant brain contexts (using a Nb specific Worniu-Gal4 driver) labeled with Dpn to visualize the Nbs. The yellow dashed line labels the central brain. Scale bar= 80 µm.

(G) Dot plot charts depicting the number of Nbs in the central brain (CB) area of $p53^{RNAi}$ in WT (red circles, n=14 lobes), in *SakOE,mad2* (green circles, n=7 lobes), and *bub3 (*blue circles*,* n=14 lobes). Each dot corresponds to one brain lobe. The line represents the mean and the error bars the SD. Statistical significance was determined using unpaired t-test. ns=not significant.

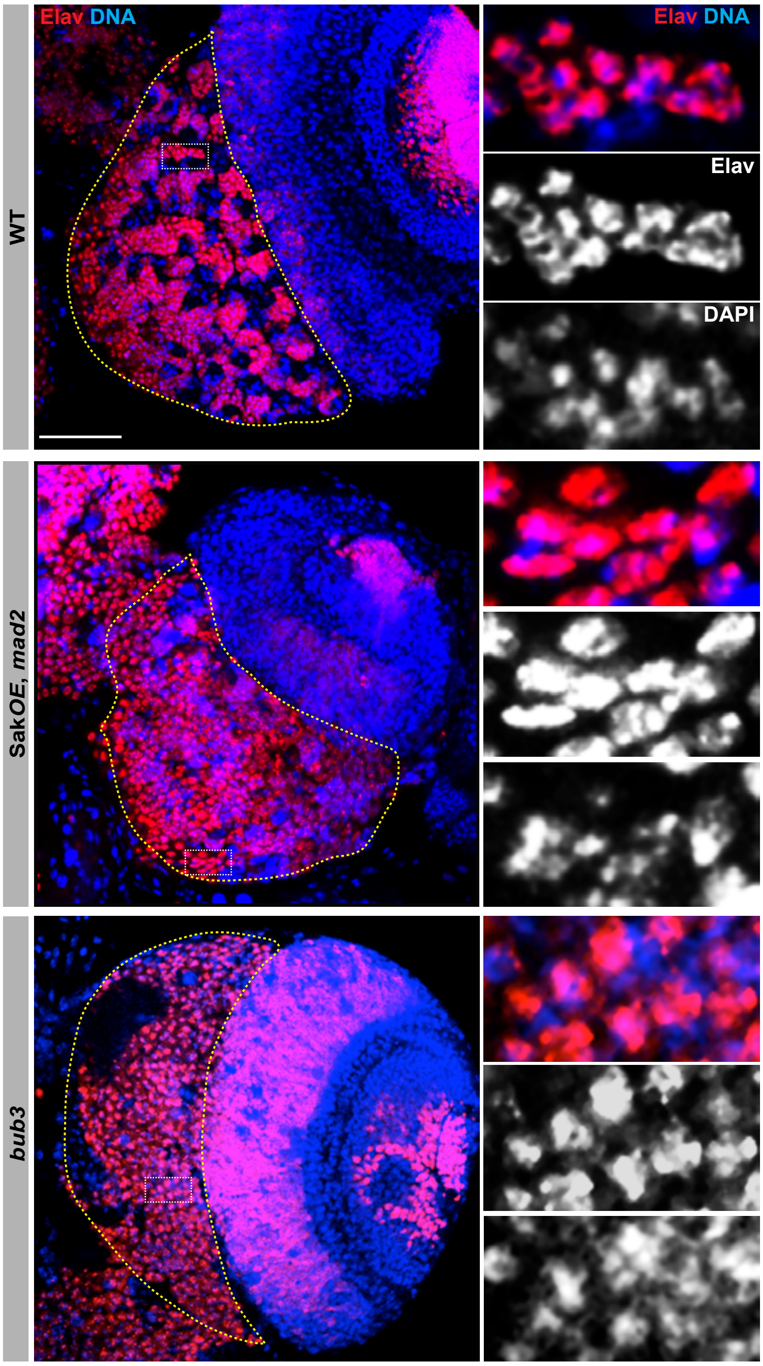

Supplementary Fig. 7- Aneuploid brains contain more neurons

Pictures of Wild type (WT, top), *SakOE,mad2* (middle) and *bub3* (bottom) brain lobes stained with Elav antibodies (shown in red in the merged panel) to label neurons. DNA is shown in blue. The yellow dashed line marks the central brain region.

Scale bar= 50 µm.

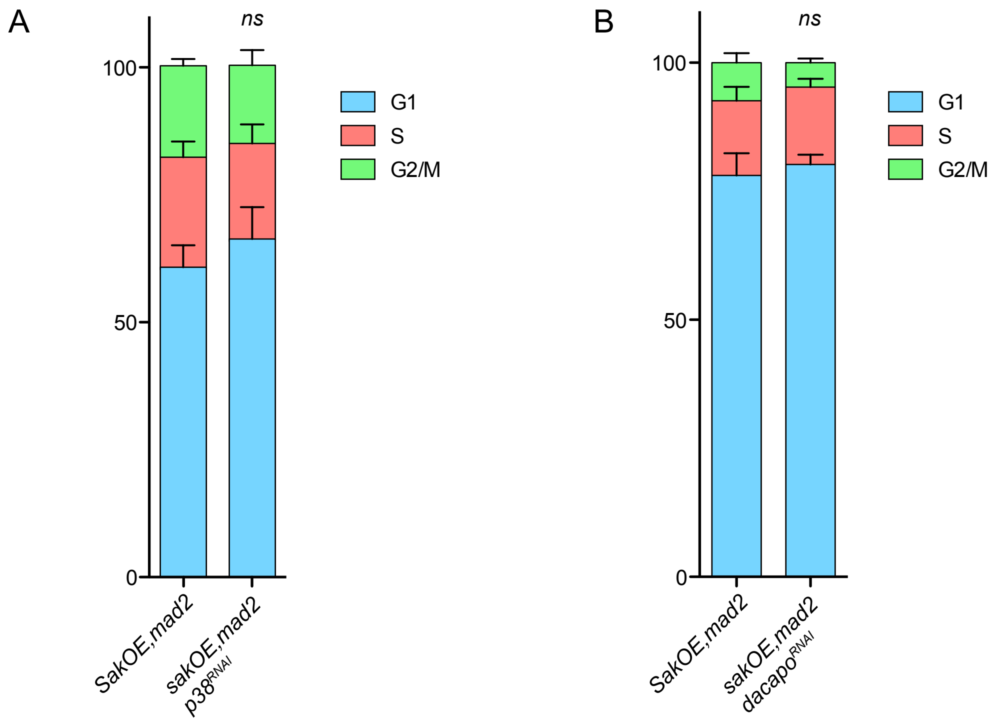

Supplementary Fig. 8- G1 extension is not p38 nor dacapo dependent

(A) Graph bar showing the quantification of cell cycle phase distribution in *SakOE,mad2* (763 Elav negative cells from 7 brain lobes) and *SakOE,mad2 wor>p38*^{RNAi} (797 cells from cells from 8 brain lobes) brains. Statistical significance was determined on each category using an unpaired t test. ns=non significant.

(B) Graph bar showing the quantification of cell cycle phase distribution in *SakOE,mad2* (281 Elav negative cells from 7 brain lobes) and *SakOE,mad2 wor>dacapo(p27)*^{RNAi} (1328 cells from cells from 7 brain lobes) brains. Statistical significance was determined on each category using an unpaired t test. ns=non significant.

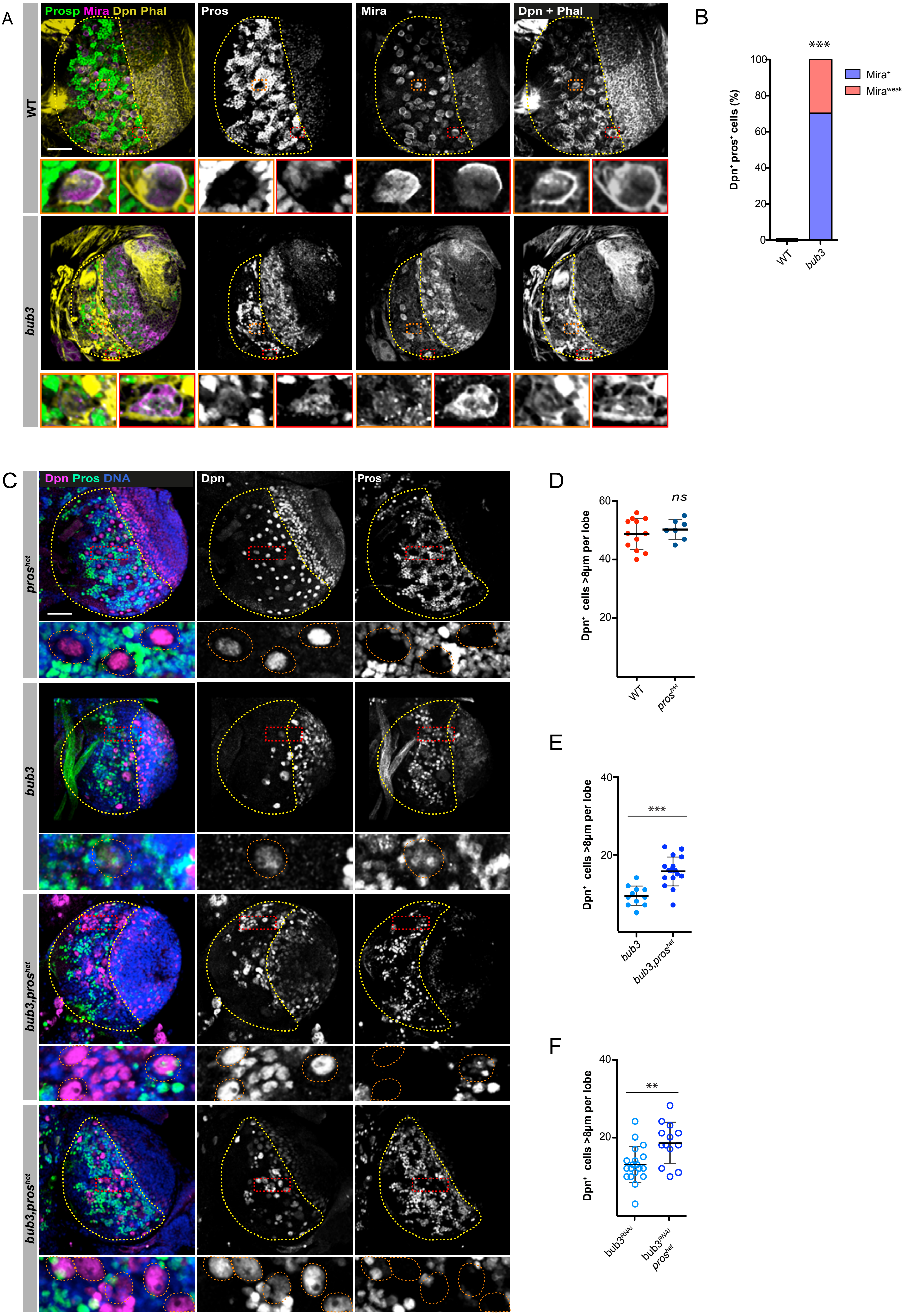

Supplementary Fig. 9- Premature differentiation is Prospero dependent

(A) Pictures of Wild type (WT) and *bub3* brain lobes labeled with Prospero (Pros) (2nd panel, shown in green in the merged panel), Miranda (Mira) (3rd panel, shown in purple in the merged panel) and Dpn and Phalloidin (right, shown in yellow in the merged panel). Scale bar=50 µm.

(B) Graph bar showing the quantification of Mira ($Mira^+$- levels similar to WT; Mira weak- lower levels similar to the cell on the left inset of *bub3* in A) in Dpn^+ $pros^+$ cells. Statistical significance was determined using a Fischer's exact test *** (p= 0.0007).

(C) Pictures of *pros* heterozygote (het) (top), *bub3* (middle) and *bub3, pros*^{het} (bottom) brain lobes stained with Dpn (shown in purple in the merged panel) and Pros (shown in green in the merged panel) antibodies to label Nbs and GMCs respectively. DNA is shown in blue. The yellow dashed line marks the central brain region. Scale bar= 50 µm.

(D) Dot plot charts depicting the number of Nbs (Dpn^+ cells >8μm) in the central brain (CB) area in WT (red circles, n=12 lobes) and *pros*^{het} (dark blue circles, n=7 lobes). Each dot corresponds to one brain lobe. The line represents the mean and the error bars the SD. Statistical significance was determined using unpaired t-test. ns=not significant.

(E) Dot plot charts depicting the number of Nbs (Dpn^+ cells >8μm) in the central brain (CB) area in *bub3* (light blue circles, n=11 lobes) and *bub3,pros*^{het} (dark blue circles, n=17 lobes). Each dot corresponds to one brain lobe. The line represents the mean and the error bars the SD. Statistical significance was determined using unpaired t-test. *** (p<0.0001).

(E') Dot plot charts depicting the number of Nbs (Dpn^+ cells >8μm) in the central brain (CB) area in *bub3*^{RNAi} (open blue circles, n=19 lobes) and *bub3*^{RNAi}*,pros*^{het} (dark blue open circles, n=13 lobes). Each dot corresponds to one brain lobe. The line represents the mean and the error bars the SD. Statistical significance was determined using unpaired t-test. ** (p=0.0038).

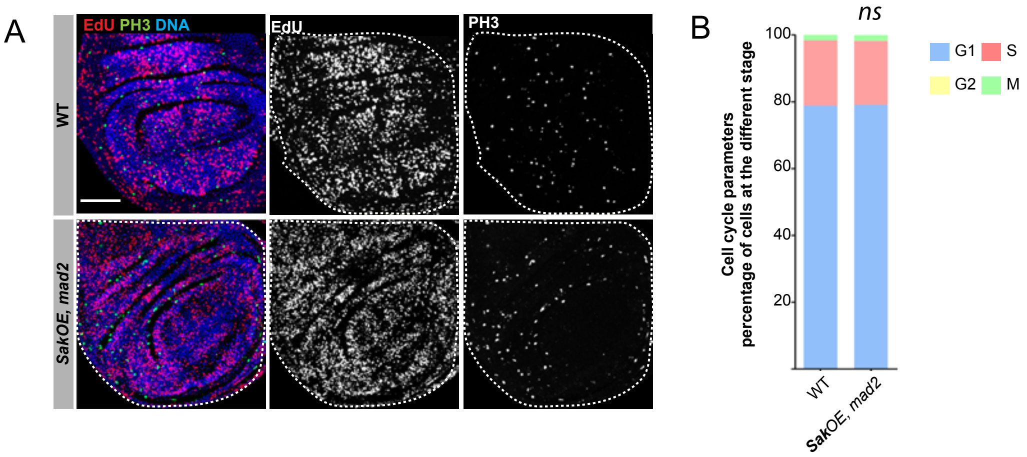

Supplementary Fig. 10- Quantification of cell cycle parameters in aneuploid wing discs

(A) Pictures of Wild type (WT) and *SakOE,mad2* third instar wing disc labeled with EdU (left, shown in red in the merged panel), phospho histone 3 (PH3) (2nd panel, shown in green in the merged panel) to label cells in S- and in M-phase respectively. DNA is shown in blue. Scale bar=50 µm.

(B) Graph bar showing the quantification of cell cycle phase distribution in WT (5841 cells from 5 wing discs) and *SakOE,mad2* (5612 cells from 3 wing discs) wing discs G1. Statistical significance was determined using an unpaired t test. ns=non significant.

Supplementary table 1- Raw data for the quantification of Dpn+ and Pros+ cells in WT, *SakOE,mad2* and *bub3* brains

	N°	Number of Prospero$^+$ cells	Number of Dpn$^+$ cells(>8µm)	Ratio Prosp$^+$/Dpn$^+$
WT	1	434	49	8,857
	2	396	24	16,500
	3	459	43	10,674
	4	466	54	8,630
	5	433	35	12,371
	6	170	13	13,077
	7	438	35	12,514
	8	456	49	9,306
	9	357	32	11,156
	10	485	36	13,472
	11	427	42	10,167
	12	261	19	13,737
	13	467	51	9,157
	14	543	40	13,575
	15	470	33	14,242
	16	598	47	12,723
	17	531	33	16,091
	18	548	47	11,660
	19	439	33	13,303
SakOE,mad2	1	487	29	16,793
	2	497	26	19,115
	3	205	14	14,643
	4	248	14	17,714
	5	170	8	21,250
	6	130	9	14,444
	7	349	27	12,926
	8	256	19	13,474
	9	334	17	19,647
	10	255	25	10,200
	11	343	23	14,913
	12	251	18	13,944
	13	261	20	13,050
	14	298	21	14,190
	15	401	28	14,321
bub3	1	127	8	15,875
	2	167	9	18,556
	3	113	7	16,143
	4	155	11	14,091
	5	157	12	13,083
	6	115	9	12,778
	7	200	14	14,286
	8	74	7	10,571
	9	94	5	18,800
	10	171	11	15,545
	11	156	10	15,600

Supplementary table 2- Raw data for the quantification of Elav+ cells and brain area in WT, *SakOE,mad2* and *bub3* brains

	N°	Number of Elav$^+$ cells	Area (μm^2)	Ratio Elav$^+$/Area
WT	1	507	39797	0,012739654
	2	393	31324	0,01254629
	3	443	35033	0,01264522
	4	238	20922	0,011375586
	5	278	30301	0,009174615
	6	331	30133	0,010984635
	7	498	57353	0,008683068
	8	545	49635	0,010980155
	9	482	38767	0,012433255
	10	405	28810	0,014057619
	11	292	25825	0,011306873
	12	457	44287	0,010319055
	13	166	36807	0,004510012
	14	435	27697	0,015705672
	15	493	29646	0,016629562
	16	392	30560	0,012827225
	17	520	50440	0,010309278
	18	380	36924	0,010291409
	19	291	22738	0,012797959
	20	565	37759	0,01496332
SakOE,mad2	1	192	12410	0,015471394
	2	134	11371	0,011784364
	3	203	14153	0,014343249
	4	228	14169	0,016091467
	5	149	11468	0,012992675
	6	361	15127	0,023864613
	7	212	16738	0,01266579
	8	271	16092	0,016840666
	9	271	13053	0,020761511
	10	232	12650	0,018339921
	11	188	14567	0,012905883
	12	224	15021	0,014912456
	13	236	14268	0,01654051
	14	203	12679	0,016010726
	15	254	18098	0,0140347
	16	239	19290	0,012389839
	17	174	20197	0,008615141
	18	176	10445	0,016850168
	19	293	16318	0,017955632
	20	210	19241	0,010914194
	21	208	18550	0,011212938
	22	305	23314	0,013082268
	23	195	11930	0,016345348
	24	281	21639	0,012985813
	25	265	25836	0,010257006
	26	329	17667	0,01862229
bub3	1	478	35332	0,013528812
	2	359	24698	0,01453559
	3	310	21764	0,014243705
	4	324	34163	0,009483945
	5	355	26338	0,013478624
	6	284	14181	0,020026796
	7	168	17210	0,009761766
	8	233	15166	0,015363313
	9	390	19505	0,019994873
	10	306	20764	0,014737045
	11	439	31915	0,013755287
	12	181	10927	0,016564473
	13	241	15422	0,015627026
	14	257	16619	0,015464228
	15	362	31118	0,011633138
	16	206	16005	0,012870978
